# Genetic Variation, Not Cell Type of Origin, Underlies the Majority of Identifiable Regulatory Differences in iPSCs

**DOI:** 10.1371/journal.pgen.1005793

**Published:** 2016-01-26

**Authors:** Courtney K. Burrows, Nicholas E. Banovich, Bryan J. Pavlovic, Kristen Patterson, Irene Gallego Romero, Jonathan K. Pritchard, Yoav Gilad

**Affiliations:** 1 Department of Human Genetics, University of Chicago, Chicago, Illinois, United States of America; 2 Howard Hughes Medical Institute, Stanford University, Stanford, California, United States of America; 3 Departments of Genetics and Biology, Stanford University, Stanford, California, United States of America; Wellcome Trust Sanger Institute, UNITED KINGDOM

## Abstract

The advent of induced pluripotent stem cells (iPSCs) revolutionized human genetics by allowing us to generate pluripotent cells from easily accessible somatic tissues. This technology can have immense implications for regenerative medicine, but iPSCs also represent a paradigm shift in the study of complex human phenotypes, including gene regulation and disease. Yet, an unresolved caveat of the iPSC model system is the extent to which reprogrammed iPSCs retain residual phenotypes from their precursor somatic cells. To directly address this issue, we used an effective study design to compare regulatory phenotypes between iPSCs derived from two types of commonly used somatic precursor cells. We find a remarkably small number of differences in DNA methylation and gene expression levels between iPSCs derived from different somatic precursors. Instead, we demonstrate genetic variation is associated with the majority of identifiable variation in DNA methylation and gene expression levels. We show that the cell type of origin only minimally affects gene expression levels and DNA methylation in iPSCs, and that genetic variation is the main driver of regulatory differences between iPSCs of different donors. Our findings suggest that studies using iPSCs should focus on additional individuals rather than clones from the same individual.

## Author Summary

Induced pluripotent stem cells (iPSCs) are a new and powerful cell type that provides scientists the ability to model complex human diseases *in vitro*. These cells can be cryopreserved and later expanded, providing a renewable source of cells from the same individual. iPSCs can be made from a variety of somatic cells in the body and many labs have created them from blood and skin cells. We asked whether the cell type of origin impacts methylation and gene expression patterns in the reprogrammed iPSCs. Our findings indicate that there are remarkably few regulatory remnants of the cell type of origin in the iPSCs. In other words, most of the variation between iPSCs can be attributed to individual genetics. Our findings suggest that studies using iPSCs should focus on obtaining additional individuals rather than additional clones from the same individual. We caution that our current findings are limited to iPSCs and further studies are needed to address the question of somatic memory in differentiated cell types.

## Introduction

Research on human subjects is limited by the availability of samples. Practical and ethical considerations dictate that functional molecular studies in humans can generally only make use of frozen post mortem tissues, a small collection of available cell lines, or easily accessible primary cell types (such as blood or skin cells). The discovery that human somatic cells can be reprogrammed into a pluripotent state [1–3] and then be differentiated [4] into multiple somatic lineages, has the potential to profoundly change human research by providing access to a wide range of cell types from practically any donor individual.

Though much progress has been made since the initial development of iPSC reprogramming technology, and human iPSCs have been used in a wide range of studies [5–8], the usefulness of iPSCs as a model system for the study of human phenotypes is still extensively debated [9–11]. The principal issue is the extent to which reprogrammed iPSCs retain epigenetic and gene expression signatures of their cell type of origin. A residual epigenetic signature of the original precursor cell in the reprogrammed iPSCs is often referred to as ‘epigenetic memory’ [12].

The common view, established by a few early studies in mice and humans, is that epigenetic memory is a significant problem in iPSCs [10,12–18]. In mice, methylation profiles in iPSCs and in the precursor somatic cells from which the iPSCs were generated were found to be more similar than expected by chance alone [12,14]. The extent of this similarity, however, could not be benchmarked against genetic diversity because the somatic cells and the iPSCs were all from genetically identical mice. In turn, methylation profiles in human iPSCs reprogrammed from different somatic cell types were found to be quite distinct from each other [15,16]. However, the somatic cells were provided by different donor individuals, hence epigenetic memory and differences due to genetic diversity were confounded.

Additionally, concerns were initially raised about residual epigenetic memory in iPSCs by studies that considered iPSCs generated using retroviral vectors [12,14–16]. Retroviral reprogramming is characterized by random integrations that vary in copy number and genomic location across lines. Furthermore, it has been shown that viral vectors commonly utilized in iPSC generation preferentially integrate into active gene bodies, strong enhancers or active promoters [19,20], this process of preferential integration into open chromatin would likely lead to a strong cell type of origin signature. In contrast to retroviral reprograming, the more recent episomal approaches to establish iPSCs are associated with much lower rates of genomic integration [21,22].

Indeed, one recent study has concluded that when properly controlling for genetic variation and using integration free methodology to establish iPSCs, the effect of cell type of origin on gene expression in iPSCs is low compared to inter-individual genetic contributions [23]. However, this study did not consider matched epigenetic markers, the supposed drivers of the suspected phenomenon of residual cell type of origin memory in reprogrammed iPSCs.

We thus designed a study to directly and effectively address this issue. We focused on two cell types that are the source for the majority of human iPSCs to date, and the most easily collected tissue samples from humans: skin fibroblasts, and blood cells. Specifically, we collected skin biopsies and blood samples from four healthy Caucasian individuals (two males and two females). Dermal fibroblasts were isolated from dissociated skin biopsies and maintained in culture until reprogramming. We isolated the buffy coat from whole blood and subsequently used Epstein–Barr virus to transform B cells into immortalized lymphoblastoid cell lines (LCLs), one of the most common cell types used in genomic studies.

## Results

**Fig 1:**
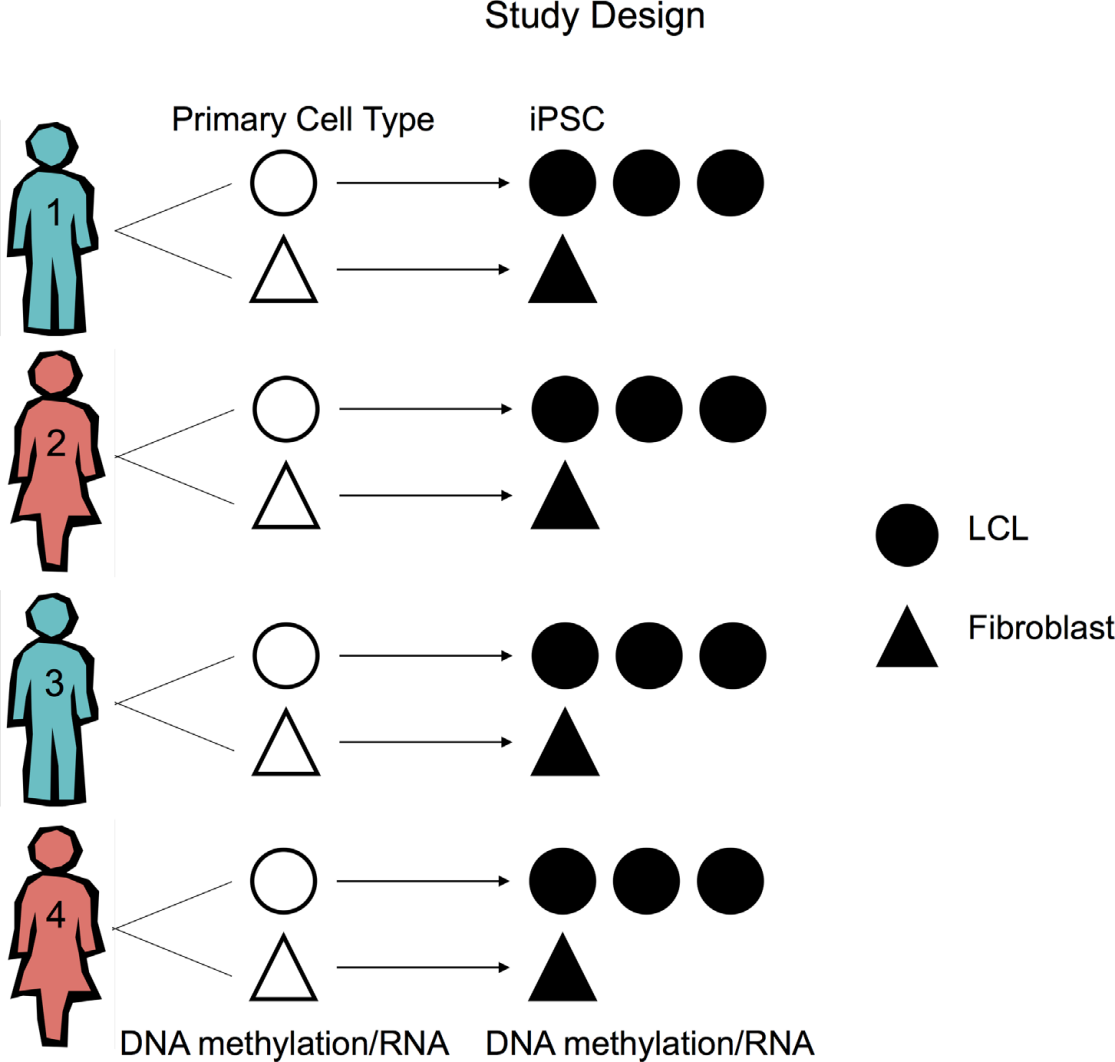
Study design. A schematic of the study design. Three independent iPSC lines were generated from LCLs and one from fibroblasts.

To determine whether cell type of origin effects gene expression and CpG methylation we reprogrammed iPSCs from two somatic tissues of four individuals. We used an episomal reprogramming approach [21] to independently generate iPSCs from the LCLs and fibroblasts of each individual, three replicates from the LCLs and one from the fibroblasts (to study epigenetic memory; Fig 1). We employed a wide range of quality control analyses and functional assays to demonstrate that all iPSCs were fully pluripotent, that they expressed endogenous, but not exogenous, pluripotency factors, that the iPSCs were free of vector integrations, and that iPSCs established from LCLs did not retain traces of integrated EBV (see methods; S1–S4 Figs).

### Cell type of origin minimally contributes to gene regulation in iPSCs

Once the quality of the iPSCs was confirmed, we extracted RNA and DNA from LCLs, fibroblasts, LCL derived iPSCs (L-iPSCs), and fibroblast derived iPSCs (F-iPSCs) from all four individuals (S1 Table). We then used the Illumina Infinium HumanMethylation450 array and the Illumina HumanHT12v4 array to measure DNA methylation and gene expression levels, respectively. Our data processing approach is described in detail in the methods. Briefly, considering the methylation data, we first excluded data from loci that were not detected either as methylated or unmethylated (no signal; detection *P* > 0.01) in more than 25% of samples. We then applied a standard background correction [24] and normalized the methylation data using SWAN [25] (S5 Fig), which accounts for the two different probe types in the platform. Finally, we performed quantile normalization (S6A and S6B Fig). Following these steps we retained methylation data from 455,910 CpGs. Considering the expression data, we first excluded probes whose genomic mapping coordinates overlapped a known common SNP. We then retained all genes that were detected as expressed in any cell type in at least three individuals (S7 Fig). We then quantile normalized the gene expression data (S6C and S6D Fig). Following these steps we retained expression data for 11,054 genes.

**Fig 2:**
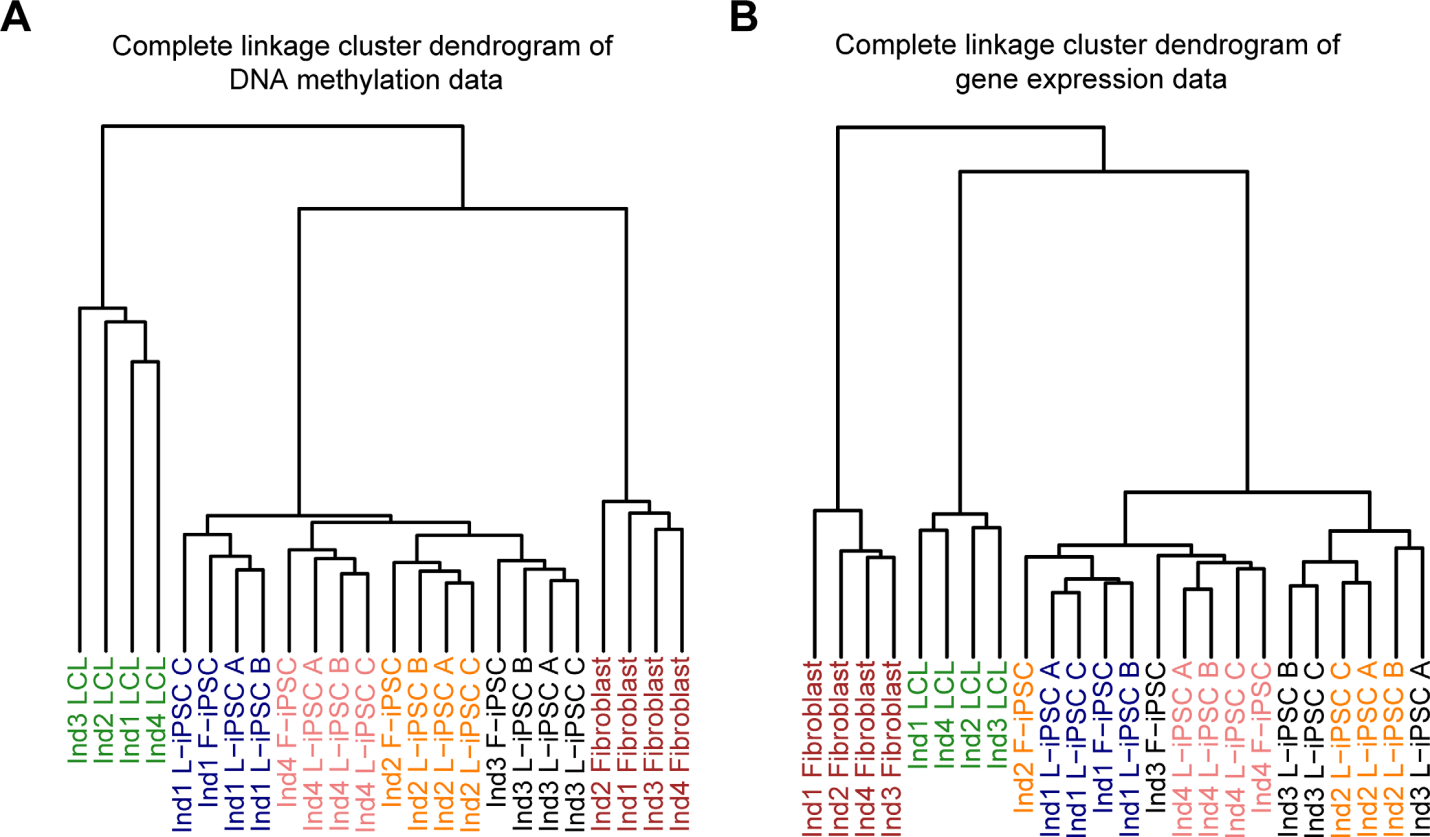
Hierarchical clustering and principal components analysis. Hierarchical clustering using the complete linkage method and Euclidean distance from autosomal loci for (a) DNA methylation data (n = 445,277 probes) and (b) gene expression data (n = 10,648 autosomal genes).

To examine overall patterns in the data, we initially performed unsupervised clustering based on Euclidean distance. As expected, using gene expression or methylation data, samples clustered based on cell type (LCLs, fibroblasts, and iPSCs) without exception. Interestingly, using the methylation data, iPSCs clustered perfectly by individual, not cell type of origin (Fig 2A). Within individual, however, data from L-iPSCs are more similar to each other than to data from F-iPSC in three of the four individual clusters. These results are consistent with a small proportion of the regulatory variation being driven by cell type of origin.

The clustering pattern is less clear when we consider the gene expression data, although the iPSCs again tend to cluster by individual more than they do by cell type of origin (Fig 2B). The property of imperfect clustering of iPSC gene expression data by individual is consistent with previous observations by Rouhani and Kumasaka et al. [23]. We believe that a possible explanation for this observation is that overall regulatory variation between iPSCs–even across individuals–is small.

Given the large number of sites interrogated (particularly on the methylation array), we also examined the clustering of iPSCs using only the top 1,000 most variable measurements across lines, similar to the approach of Kim et al. 2011 [16]. Our clustering remained largely unchanged using this subset of variable sites for both methylation data (S8A Fig) and expression data (S8B Fig). Clustering based on pairwise Pearson correlations rather than Euclidian distance produced nearly identical results (S8C–S8F Fig). We also examined patterns in the data using principal components analysis (PCA; S9 Fig) The results from the PCA are not as easily interpretable as those from the clustering analysis, but it is clear that the major components of variation are not driven by cell type of origin.

### Little evidence of widespread epigenetic memory in iPSCs

**Fig 3:**
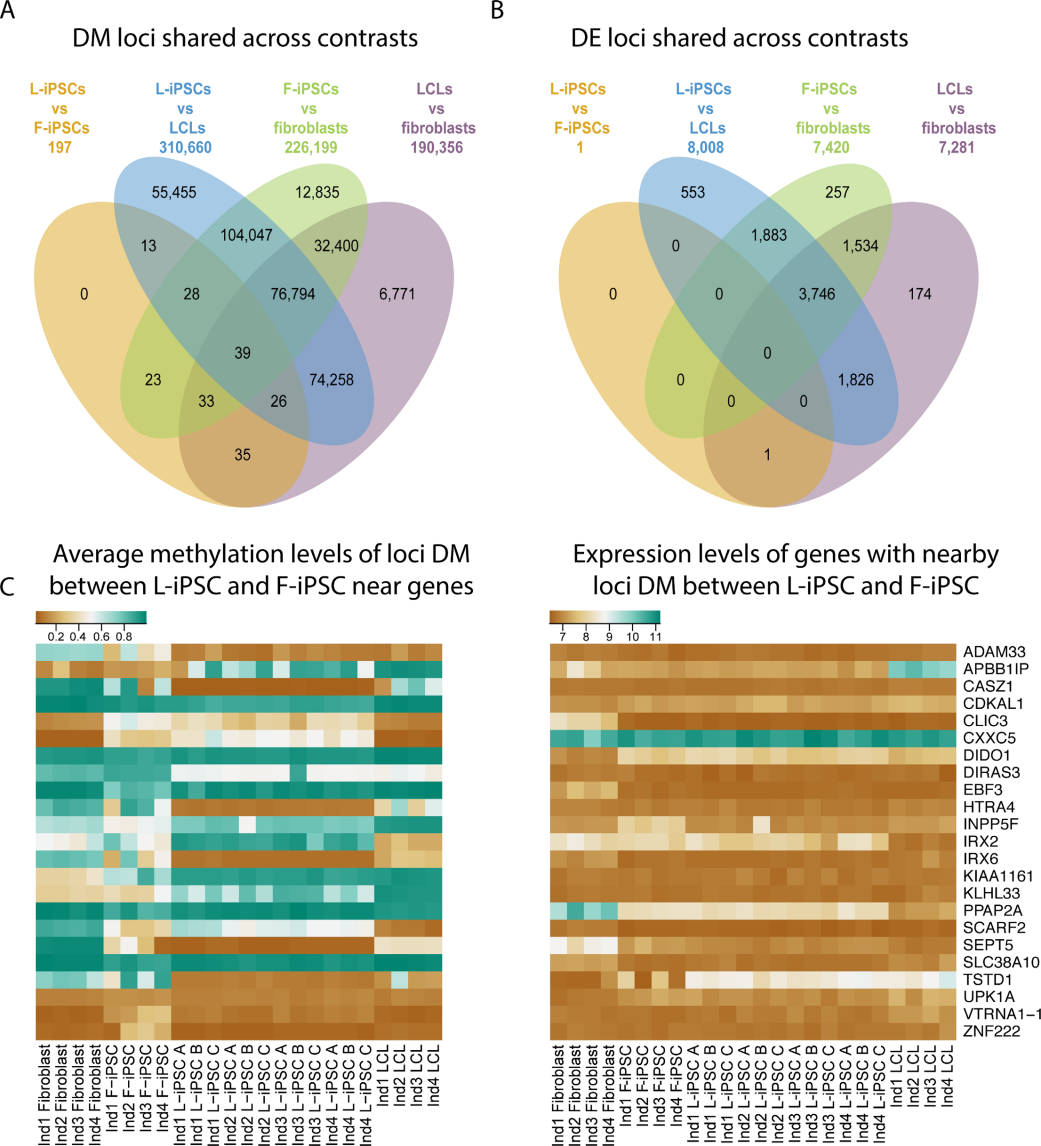
Differential methylation and gene expression between the four cell types (L-iPSC, F-iPSC, LCLs and fibroblasts). (a) A Venn diagram of differentially methylated (DM) loci (FDR of 5%) overlapping between different contrasts. (b) Venn diagram of differentially expressed (DE) genes (FDR of 5%) overlapping between different contrasts. (c) Heatmaps of the DNA methylation and gene expression levels where each row corresponds to a gene (labeled on the right). DNA methylation levels represent the average of all loci DM between L-iPSCs and F-iPSCs nearby the corresponding gene.

We next considered methylation and expression patterns at individual loci and genes, respectively. We first focused on differences in CpG methylation between the cell types. Using limma [26] (see methods), we identified 190,356 differentially methylated (DM) CpG loci between LCLs and fibroblasts (FDR of 5%). Similarly, we identified 310,660 DM CpGs between LCLs and L-iPSCs and 226,199 DM loci between fibroblasts and F-iPSCs (Fig 3A). In contrast, at the same FDR, we only classified 197 CpG loci (0.04% of the total sites tested; S10 Fig) as DM between L-iPSCs and F-iPSCs (S2A–S2D Table). Moreover, the 197 DM loci were not all independent; they clustered into 53 genomic regions, 37 of which are located near or within annotated genes. Of these 37 genes, 24 had measurable gene expression data (Fig 3C).

The observation of small number of significant DMs associated with cell type of origin does not preclude a persistent but small difference between the epigenetic landscapes of L-iPSCs and F-iPSCs. We therefore asked, for each CpG classified as DM between LCLs and fibroblasts, whether the sign of the mean methylation difference between L-iPSCs and F-iPSCs is the same as the sign of the mean difference between the cell types of origin. We found a slight but significant enrichment of a consistent sign (50.5% of the loci; binomial test; *P* < 10^−6^) in these two contrasts. This observation confirms that while epigenetic memory in iPSCs can be detected, the magnitude of such effect is small.

Of the 197 DM loci between L-iPSCs and F-iPSCs, 133 loci were also DM between LCLs and fibroblasts (a highly significant overlap*; χ*^*2*^ test; *P < 10*^*−15*^). Moreover, 122 of these 133 DM loci showed a difference in methylation between LCLs and fibroblasts that was in the same direction as the one seen between L-iPSCs and F-iPSCs (sign test; *P < 10*^*−15*^). In principle, these observations support the idea of epigenetic memory, namely that a subset of epigenetic differences between the somatic cells persists in the reprogrammed iPSCs. Yet our results indicate that epigenetic memory persists in a remarkably small number of loci.

### A single DE gene between F-iPSCs and L-iPSCs

**Fig 4:**
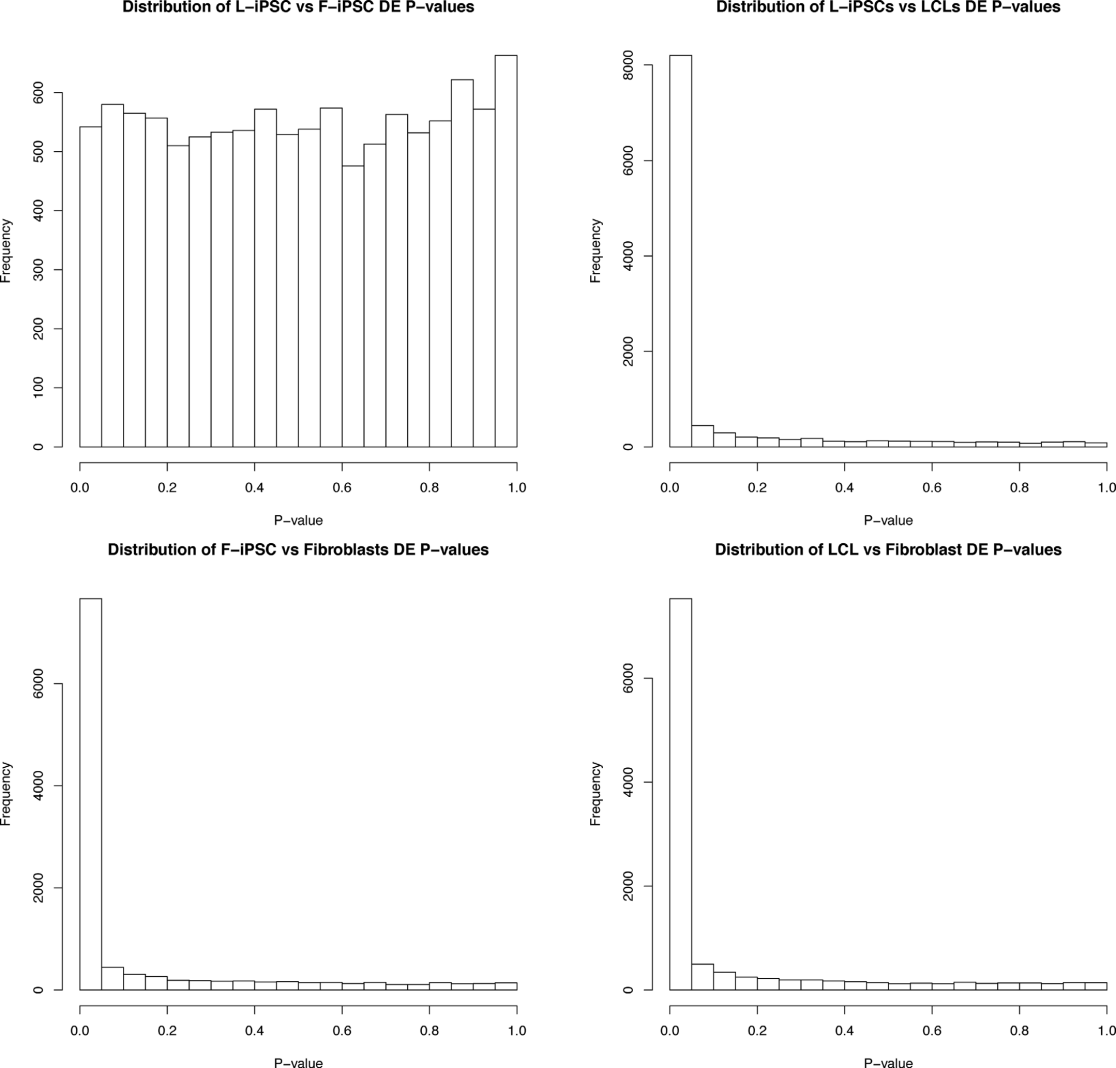
Histograms of P-values from DE tests. Histograms of *P*-Values from differential expression analysis.

We turned our attention to the gene expression data. We again used limma to identify (at an FDR of 5%) 7,281 differentially expressed (DE) genes between LCLs and fibroblasts, 8,008 DE genes between LCLs and L-iPSCs, and 7,420 DE genes between fibroblasts and F-iPSCs (Fig 3B). In contrast, at the same FDR, we classified only a single gene (*TSTD1*) as DE between L-iPSCs and F-iPSCs. These results are consistent with recent observations [23]. More generally, we found nearly no evidence for departure from a null model of no differences in gene expression levels between L-iPSCs and F-iPSCs (Fig 4, S11 Fig; S3A–S3D Table). We proceeded by performing a sign test, considering the sign of the mean gene expression difference between L-iPSCs and F-iPSCs in genes that were classified as DE between LCLs and iPSCs. We found fewer consistent signs than expected by chance alone (47.8%; binomial test: *P* = 10^−4^).

The single DE gene between L-iPSCs and F-iPSCs, *TSTD1* (*P = 6*.*28 x 10*^*−7*^; FDR 0.69%), is also DE between the LCLs and fibroblasts precursor cells. Moreover, 11 of 19 CpG sites that are located near the *TSTD1* gene, and are assayed by the methylation array, are among the 197 DM loci between L-iPSCs and F-iPSCs. We observed a decreased fold change of *TSTD1* expression when comparing between LCLs and fibroblasts (log2 fold change of 2.06) and L-iPSCs and F-iPSCs (log2 fold change of 1.34). This may be a case of epigenetic memory that maintains a gene expression residual difference, but it appears to be the only such case in our data. We found no evidence that any of the other DM loci are associated with gene expression differences between L-iPSCs and F-iPSCs (Fig 3C). This is true even when we conservatively accounted for multiple tests by only considering the number of tests that involved genes that are associated with DM loci between L-iPSCs and F-iPSCs (S11 Fig).

**Fig 5:**
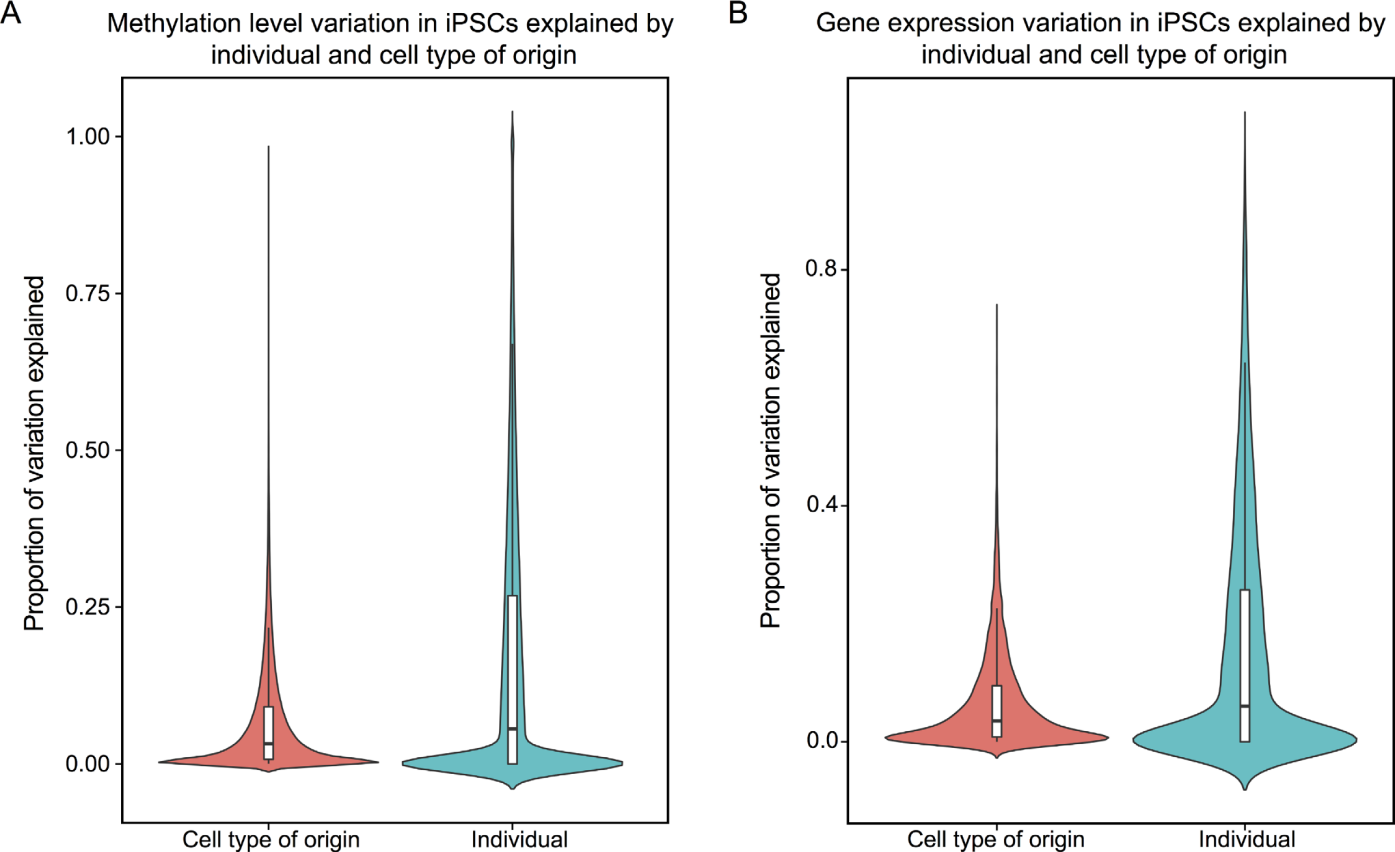
Contribution of individual differences versus cell type of origin to methylation and expression levels. Estimated contribution of inter-individual differences and cell type of origin effects on variation in (a) methylation and (b) gene expression levels from a linear mixed effect model. There is a significant difference in the mean proportion of variation explained by individual and cell type of origin (*P* < 10^−15^).

Our observations indicate that remarkably little residual memory of the precursor somatic cell affects gene expression and methylation patterns in the reprogrammed iPSCs. To formally evaluate this we estimated the contribution of inter-individual differences and cell type of origin effects on variation in methylation and gene expression levels (see methods). The mean proportion of variance explained by donor individual is 16.2% and 15.5%, for the methylation and expression data, respectively; while the mean proportion of variance explained by cell type of origin is 6.6% and 6.7%, respectively (T-test; *P* < 10^−15^; KS test *P* < 10^−15^; Fig 5). Interestingly, when we focus on gene and CpGs whose expression and methylation levels in LCLs were previously associated with genetic variation (eQTLs and meQTLs, respectively), the mean proportion of variance explained by donor individual is significantly higher (21.2% and 19.9%, for the methylation and expression data, respectively; T-test *P* < 10^−15^; KS test *P* < 10^−15^; S13 Fig), while the mean proportion of variation explained by cell type of origin is roughly similar (6.28% and 6.34% for methylation and expression data, respectively).

## Discussion

To date, the common view is that iPSCs derived from somatic cells retain robust epigenetic traces of the precursor cells [10,12–17,24]. Yet, in our data, a remarkably small amount of the observed regulatory variation in iPSCs is driven by cell type of origin. Our observations are consistent with genetic background being a major driver of regulatory variation in iPSCs.

While our results challenge the common view that epigenetic memory is prevalent in iPSCs, a careful examination of the literature suggests that our data are in fact consistent with previous studies, though our interpretation is not. The principal difference between previous studies and ours is that we were able to benchmark epigenetic memory against other sources of variation. Previous studies either characterized iPSCs from a single individual [12,14], or were not able to distinguish between genetic and cell type of origin effects [15,16]. For example, though Kim et al. [16] reported a similar number of DM loci (137–370) between iPSCs derived from different cell types as we observed in our study, Kim et al. interpreted their observation as evidence for a marked effect of the donor cells. Yet, our observation that DNA methylation is quite homogenous across all iPSCs (both within replicates and between L-iPSCs and F-iPSCs; S8C and S8D Fig), is not in disagreement with the observations of Kim et al.

Indeed, our study explicitly models the contribution of genetic background to variation in DNA methylation levels in iPSCs. When we consider DNA methylation in the context of variation explained by inter-individual differences, we find a remarkably small effect associated with cell type of origin. Moreover, even unsupervised clustering (based on either DNA methylation or gene expression data) indicated that samples largely clustered by individual. We found little evidence of clustering by cell type of origin. When we turned our attention to individual loci, only 197 (0.043%) tested CpGs were classified as DM between L-iPSCs and F-iPSCs, compared with 190,356 (41.7%) loci that were classified as DM between LCLs and fibroblasts.

Our observation that only a handful of DM sites may drive regulatory differences between iPSCs from different origins is consistent with recent work by Rouhani and Kumasaka et al. [23] where a similar study design was employed examining only gene expression levels. Indeed, as in Kumasaka et al., we found that individual genetic background captures a much larger proportion of gene regulatory variation than cell type of origin using both the DNA methylation and gene expression data.

Future work needs to address additional pertinent questions. First, our study was limited to methylation and gene expression levels in iPSCs. Future studies should focus on additional epigenetic and regulatory markers. Second, we focused on regulatory differences between iPSCs, but did not study differentiated cell types. This needs to be addressed in the future because the degree to which iPSCs retain regulatory signatures of their cell type of origin ultimately is expected to influence the extent to which iPSCs can be used as a model system for studying complex traits in differentiated cell types.

In conclusion, our study demonstrated that when accounting for individual, the impact of cell type of origin on DNA methylation and gene expression in iPSCs is limited to a small number of CpGs, which cluster into an even smaller number of genomic loci, and a single gene, with almost no detectable influence genome-wide. Our observations further confirm the usefulness of iPSCs for genetic studies regardless of the original somatic cell type. The high correlation of DNA methylation and gene expression levels (S8C and S8D Fig) between individuals, demonstrate the faithfulness of the model, though as we pointed out–similar studies in differentiate cells are required to generalize these conclusions. While cell type of origin should continue to be carefully documented, our data also suggest that future studies should focus on collecting more individuals rather than establishing multiple iPSC clones from the same individual.

## Materials and Methods

### Isolation and culture of fibroblasts and LCLs

Skin punch biopsies and blood were collected from the same individual within 20 minutes under University of Chicago IRB protocol 11–0524 (samples from four individuals were collected over three collection dates; samples from individuals 3 and 4 were collected on the same date). Skin and blood samples from an individual were processed at the same time (S1 Table). Fibroblast isolation and culture was conducted using the approach described in detail in Gallego Romero et al [27]. Briefly, skin punch biopsies (3mm) were digested using 0.5% collagenase B (Roche), isolated fibroblasts were cultured in DMEM (Life Technologies) supplemented with 10% fetal bovine serum (FBS; JR Scientific), 0.1mM NEAA, 2mM GlutaMAX (both from Life Technologies), 1% penicillin/streptomycin (Fisher), 64mg/L L-ascorbic acid 2-phosphate sesquimagnesium salt hydrate (Santa Cruz Biotechnology), at 5% CO_2_ and 5% O_2_.

All other cell culture was performed at 5% CO_2_ and atmospheric O_2_. For LCL generation, whole blood was drawn (within 20 minutes of obtaining skin punch biopsies) into two 8.5mL glass yellow top tubes (Acid Citrate Dextrose Solution A tubes; BD). Blood tubes were stored at room temperature and processed within 12 hours of collection. To isolate lymphocytes, we diluted whole blood with an equal amount of RPMI 1640 (Corning), diluted blood was slowly layered onto Ficoll-Paque (GE Lifescience) in 50 mL centrifuge tubes. This gradient was centrifuged at 1700 rpm for 30 minutes without acceleration or braking. Leukocytes and platelets formed a white band at the interface between the blood plasma and the Ficoll (called the buffy coat). We collected the buffy coat using a Pastette and to that added 10mL of PBS. The collected buffy coat was then washed three times with PBS.

For EBV transformation, 4 x 10^6^ fresh lymphocytes collected as described above were resuspended in a total of 4.5 ml of RPMI 1640 culture medium (Corning) containing 20% FBS and 1:100 phytohemagglutinin (PHA-M; LifeTechnologies) and transferred to a T-25 flask. EBV supernatant produced by the B95-8 cell lines (provided by the Ober lab) was added at 1:10 to the culture flask. Cells were left undisturbed for three to five days before adding fresh media. Flasks were subsequently examined weekly for changes in cell growth as indicated by acidic pH (yellow color) and the appearance of clumps of cells growing in suspension. Once growth was established (21–35 days), cells were diluted or split to several flasks. When the cell density reached 8 x 10^5^ to 1 x 10^6^ cells per mL they were cryopreserved at a density of 10 x 10^6^ cells per ml of freezing media in cryovials. All LCLs using this study were transformed with the same lot of EBV supernatant.

### Episomally-reprogrammed iPSCs

To establish iPSCs we transfected LCLs (Amaxa Nucleofector Technology; Lonza) and fibroblasts (Neon Transfection System; Life Technologies) with oriP/EBNA1 PCXLE based episomal plasmids that containing the genes *OCT3/4*, *SOX2*, *KLF4*, *L-MYC*, *LIN28*, and an shRNA against *p53* [21]. We supplemented these plasmids with an *in vitro*-transcribed EBNA1 mRNA transcript to promote exogenous vector retention following electroporation of the episomal vector [28,29]. Fibroblasts from all individuals were reprogrammed in two batches (see details in S1 Table). LCLs were reprogrammed in four batches (S1 Table). The first three batches contained LCLs from all four individuals. Individual 4 failed reprogramming in batches one and three. A final fourth batch was therefore done with only individual 4 (replicates A and C; S1 Table). We plated a range of 10,000–40,000 transfected cells per well in a 6-well plate. Within 21 days colonies were visible and manually passaged onto a fresh plate of irradiated CF1 mouse embryonic fibroblasts (MEF). We passaged these new iPSC colonies on MEF in hESC media (DMEM/F12 (Corning) supplemented with 20% KOSR (LifeTechnologies), 0.1mM NEAA, 2mM GlutaMAX, 1% Pen/Strep, 0.1% 2-Mercaptoethanol (LifeTechnologies)). Fibroblast derived iPSCs were supplemented with 100ng/mL human basic fibroblast growth factor, versus 25ng/mL for LCL derived iPSCs; all other culture conditions were identical. After 10 passages of growth we transitioned the cultures to feeder-free conditions and cultured them for an additional three passages before collecting cell pellets for analysis. Feeder-free cultures were grown using 0.01mg/cm^2^ (1:100) hESC-grade Matrigel (BD Sciences) and Essential 8 media (LifeTechnologies). Passaging was done using DPBS supplemented with 0.5mM EDTA. All RNA and DNA were isolated using Zymo dual extraction kits (Zymo Research) with a DNase treatment during RNA extraction (Qiagen).

### Characterization of iPSCs

All iPSC lines were characterized as described previously [27]. Briefly, we initially confirmed pluripotency using PluriTest [30], a classifier that assigns samples a pluripotency score and novelty score based on genome-wide gene expression data. All samples were classified as pluripotent and had a low novelty score (S1 Fig). We next performed qPCR using 1 μg of total RNA, converted to cDNA, from all samples to confirm the endogenous expression of pluripotency genes: *OCT3/4*, *NANOG*, and *SOX2* (S2A–S2C Fig). Additionally, we tested for the presence and expression of the EBV gene *EBNA-1* using PCR (primers and cycling conditions can be found in S5 Table) (S2D and S3 Figs). We tested all samples for both genomic integrations and vector-based EBV. We did this using primers designed to amplify the *EBNA-1* segment found in both the episomal vectors and the EBV used to transform LCLs. If the cell was positive (a single positive case was found: Ind4 F-iPSC), we further tested the origin of the EBV (genomic or episomal) using primers specific to the *LMP-2A* gene found in EBV or part of the sequence specific to the episomal plasmid (S3 Fig). Finally, we confirmed the ability of all iPSC lines to differentiate into the three main germ layers using the embryoid body (EB) assay. The EBs were imaged for the presence of all three germ layers (S4 Fig). It should also be noted that gene expression and DNA methylation levels are extremely similar between iPSC lines. This relative homogeneity further demonstrates the quality of our iPSC lines. In summary, all iPSC lines established in this study showed expression of pluripotent genes quantified by qPCR, generated EBs for all three germ layers, and were classified as pluripotent based on PluriTest.

### Processing of methylation array

Extracted DNA was bisulphite-converted and hybridized to the Infinium HumanMethylation450 BeadChip (Illumina) at the University of Chicago Functional Genomics facility. To validate the array probe specificity, probe sequences were mapped to an *in silico* bisulfite-converted genome using the Bismark aligner [31]. Only probes that mapped uniquely to the human genome were retained (n = 459,221). We further removed data from probes associated with low signal (detection *P-value > 0*.*01*) in more than 25% of samples (retained data from n = 455,910 loci). Raw output from the array (IDAT files) were processed using the minfi package [24] in R.

We performed standard background correction as suggested by Illumina [24], and corrected for the different distribution of the two probe types on the array using SWAN [25] (S5 Fig). Additionally, we quantile normalized the red and green color channels (corresponding to methylated and unmethylated signal respectively) separately (S6A and S6B Fig). To calculate methylation levels (reported as β-values) we divided the methylated signal by the total signal from both channels. β-values were considered estimates of the fraction of alleles methylated at that particular locus in the entire cell population.

### Processing of expression arrays

RNA quality was confirmed by quantifying sample’s RNA Integrity Number (RIN) on an Agilent 2100 Bioanalyzer (Agilent Technologies). All samples had a RIN of 10. The extracted RNA from all samples was hybridized to the Illumina HT12v4 Expression BeadChip array (Illumina) at the University of Chicago Functional Genomics facility. Sample processing was performed using the lumi package in R [32]. We excluded data from a subset of probes prior to our analysis: First, we mapped the probe sequences to the human genome hg19 and kept only those with a quality score of 37, indicative of unambiguous mapping (n = 40,198; note that we also explicitly pre-filtered the 5,587 probes which were annotated as spanning exon-exon junctions to avoid mapping errors). Second, we downloaded the HapMap CEU SNPs (http://hapmap.ncbi.nlm.nih.gov/downloads/genotypes/2010-08_phaseII+III/forward/) and converted their coordinates from hg18 to hg19 using the UCSC liftOver utility [33]. We retained only those probes that did not overlap any SNP with a minor allele frequency greater than 5% (n = 34,508). Third, we converted the Illumina probe IDs to Ensembl gene IDs using the R/Bioconductor package biomaRt [34] and retained only those probes that are associated with exactly one Ensembl gene ID (Ensembl 75—Feb 2014; n = 22,032). The full pipeline was implemented using the Python package Snakemake [35]. We defined a gene as expressed in a given sample if at least one probe mapping to it had a detection *P-value* < 0.05. In the case of L-iPSCs, we defined a gene as expressed in an individual if any associated probes had a detection *P-value* < 0.05 in at least one biological replicate. Using these criteria, we identified all genes expressed in at least three individuals in at least one cell type (S7 Fig; n = 14,111 probes associated with 11,054 annotated genes). In the case that multiple expressed probes were associated with the same ENSEMBL gene (n = 3,057), we only retained data from the 3'-most detected probe. Following these filtration steps, we obtained estimates of expression levels in all samples across 11,054 genes. Data from the 11,054 genes were quantile normalized using the lumiExpresso function in lumi [32] (S6C and S6D Fig).

### Unsupervised hierarchical clustering and heatmaps

Only data from autosomal probes were retained for the hierarchical clustering analyses in order to reduce bias towards clustering by individual or sex (n = 10,648 expression, and n = 445,277 methylation). We calculated a matrix of pairwise Euclidean distances between samples from the methylation and expression data separately. From these matrices we performed hierarchical clustering analyzing using the complete linkage method as implemented in the R function hclust. The observed dendrograms remained consistent regardless of the linkage method chosen (complete, single, or average). The 1,000 most variable loci were defined by taking the loci with the highest variance in iPSCs. Clustering based on the 1,000 most variable probes were processed in an identical manner as above. Heatmaps were generated from matrices of pairwise Pearson correlations between samples using data from autosomes and sex chromosomes.

### Analysis of differences in gene expression and methylation levels

Data from probes on both autosomes and sex chromosomes were included in this analysis, given that individuals were balanced across cell types (n = 455,910 CpGs; n = 11,054 genes). Additionally, we anticipated that sites on the sex chromosomes may be particularly sensitive to mis-regulation during reprogramming [36]. Differential expression and methylation analyses were performed using linear modeling and empirical Bayes methods as implemented in the limma package [26]. We tested for differential methylation and expression, using locus-specific models, between L-iPSCs and F-iPSCs; L-iPSCs and LCLs; F-iPSCs and fibroblasts; and between fibroblasts and LCLs. We considered a locus DM or DE at an FDR < 5% (Benjamini Hochberg). We also tested for DE genes between L-iPSCs and F-iPSCs using only genes that were classified as DE between L-iPSCs and LCLs; F-iPSCs and fibroblasts; and LCLs and fibroblasts (S11 Fig). We estimated FDRs separately each time we considered only subsets of the data.

Due to the imbalance of L-iPSC samples to F-iPSC samples we repeated our analyses using data from a reduced set of samples. Namely, we randomly sampled a single replicate of the L-iPSC from each individual. As expected, reducing the number of L-iPSC samples greatly reduces the number of loci classified as DM between L-iPSCs and F-iPSCs as well as between L-iPSCs and LCLs. However, the number of DM loci was reduced across all other contrasts as limma models the entire matrix together (S12 Fig). Interestingly, we found that different combinations of replicates yielded DE genes other than *TSTD1*. Therefore, we sampled all possible combinations and overall, found six genes that were classified as DE (FDR 5%; see S4 Table) in at least one of the combinations of reduced samples. Of note, we never classify *TSTD1* as DE (FDR 5%) in the reduced data set. The most common DE gene, *INPP5F*, is the only gene that also has nearby DM CpGs (five of the 25 nearby loci). Additionally, in the full model, *INPP5F* has the second lowest *P value* (uncorrected *P = 6*.*84 x 10*^*−5*^; FDR 38%). However, *INPP5F* was not DE between LCLs and fibroblasts, but was DE between LCLs and L-iPSCs and also fibroblasts and F-iPSCs (S3A–S3D Table; Fig 3C).

### Enrichment of DM loci in regulatory and genomic features

We employed two strategies to identify enrichments of DM loci between L-iPSCs and F-iPSCs in regulatory features. First, we used the regulatory states defined by Ernst et al. [37]. We tested for enrichments in all regulatory categories using a χ-square test comparing the number DM loci and total probes within each regulatory class to the number DM loci and total probes outside the regulatory class. We found no significant enrichment for any of the defined regulatory states.

Next, we used the UCSC_RefGene_Group annotation as supplied by Illumina. These annotations detail the location of probes in relation to genes (1st Exon, 3' UTR, 5' UTR, Gene Body, within 1.5kb of a TSS or within 200bp of a TSS). We identified significant enrichments of DM loci within 1.5kb of a TSS and gene bodies. However, there are six probes classified as both within a gene body and within 1.5kb of a TSS. We chose to report both results because it is difficult to deconvolute these categories.

We also considered the position of DM loci in relation to genes. The annotations were defined by Illumina. We were able to identify 37 genes associated with DM loci, but we only had corresponding gene expression data for 24 of these genes. We attempted to identify signals of enrichment in DE levels between L-iPSCs and F-iPSCs in these 24 genes. To this end, we compared the log fold changes in gene expression between L-iPSCs and F-iPSCs from genes with nearby DM loci between L-iPSCs and F-iPSCs to 10,000 random samplings of log fold change in expression between L-iPSCs and F-iPSCs from all genes and found no enrichment for increased log fold changes.

### Proportion of variance explained

To estimate the proportion of variance explained by individual and cell type of origin we performed a linear mixed model with a fixed effect for cell type of origin and a random effect for individual. Only data from autosomes were included in this analysis so that the results would not be biased toward differences in individuals (n = 10,648 expression, and n = 445,277 methylation). To calculate the proportion of variance explained we divided the variance components of each term by the total variance in gene expression (Fig 5). When focusing on CpGs and genes with previously identified genetic associations (eQTLs and meQTLs, respectively) we used genes with at least one eQTL identified by Lappalainen et al. 2013 [38] and CpGs with at least one meQTL identified by Banovich et al. 2014 [39] (S11 Fig).

### Accession numbers

The expression and methylation data sets supporting the results of this article are available in the Gene Expression Omnibus (GEO) under accession GSE65079 (http://www.ncbi.nlm.nih.gov/geo/query/acc.cgi?acc=GSE65079).

### Ethics, consent and permissions

All individuals consented to study participation under University of Chicago IRB protocol 11–0524.

## Supporting Information

S1 Fig Quality control of iPSCs. iPSC lines QC—PluriTest pluriscore results for all samples, showing all iPSC samples fall within the pluripotent threshold (red dashed lines). Additionally, all primary tissue samples fall within the non-iPSC cell type classification (blue dashed lines).
(PDF)

S2 Fig Quality control of iPSC lines. iPSC lines QC—Quantitative PCR (qPCR) of pluripotency genes (a) *OCT3/4*, (b) *NANOG*, and (c) *SOX2* normalized on randomly selected Ind3 L-iPSC C. Relative expression is the RQ value with respect to *GAPDH* expression, with error bars representing the calculated min and max RQ value. All iPSC lines show endogenous expression of these pluripotency genes. (d) Expression of *EBNA-1*, a required viral gene of Epstein-Barr virus (EBV), normalized on randomly selected Ind3 LCL. *EBNA-1* expression could stem from either the reprogramming vectors or, in LCLs and L-iPSCs, expression of integrated genomic EBV. Ind4 F-iPSC shows low expression of EBNA-1 due to low retention of reprogramming vectors as confirmed in Supplementary Fig 3. This sample is kept for data analysis because all other QC measures are met and the sample is not an outlier in overall gene expression or DNA methylation.
(PDF)

S3 Fig Quality control of iPSCs. (a) PCR on DNA for presence or absence of EBV, both integrated and non-integrated (reprogramming vector based). All four LCLs showed the presence of EBV along with one iPSC line, Ind4 F-iPSC. Additional banding in the images is due to RNA in the sample. These five samples, highlighted by a red box, were taken forward for two additional PCRs. First, the five samples were tested for the presence of the reprogramming vectors (b), of which only Ind4 F-iPSC was positive. Lastly, the five samples were tested for EBV based on the presence of the *LMP-2A* sequence (c; an EBV gene not found on the reprogramming vector). All LCLs were positive for EBV, and the iPSC sample was not.
(PDF)

S4 Fig Quality control of iPSCs. iPSC lines QC—Embryoid body (EB) formation from iPSC lines to validate the ability to differentiate into all three germ layers. The leftmost column (a) shows EBs stained with Nestin, a cytoplasmic stain for ectoderm in green and MAP2, a cytoplasmic stain for ectoderm in red. The center column (b) shows EBs stained with SMA, a cytoplasmic stain for mesoderm in green and again for MAP2 in red. The rightmost column (c) shows EBs stained with AFP, a cytoplasmic stain for endoderm in green and HNF3β, a nuclear stain for endoderm in red. All iPSC lines generated showed the ability to differentiate into all three germ layers. All imaging was done at 10x magnification and nuclei were stained blue with Hoechst.
(PDF)

S5 Fig DNA methylation density plots. Representative density plots of DNA methylation levels separated by type I and type II probes before and after SWAN Normalization.
(PDF)

S6 Fig Array data normalization. Methylation levels (Beta) (a) pre- and (b) post- quantile normalization. Quantile normalization was performed independently on the red and green color channels. Gene expression data (c) pre- and (d) post- quantile normalization.
(PDF)

S7 Fig Probe inclusion scheme. For 22,032 probes we defined a gene as expressed in a given sample if at least one probe mapping to it had a detection P-Value < 0.05. In the case of L-iPSCs, we defined a gene as expressed in an individual if any associated probes had a detection P-Value < 0.05 in at least one biological replicate. Using these criteria, we identified all genes expressed in at least three individuals in at least one cell type (n = 14,111 probes, associated with 11,054 genes).
(PDF)

S8 Fig Hierarchical clustering. Hierarchical clustering using the complete linkage method and Euclidean distance from the 1,000 most variable autosomal iPSC loci for (a) methylation data and (b) expression data. Heatmap showing pairwise Pearson correlations between all samples for all loci (autosomes and sex chromosomes) (c) methylation data and (d) gene expression data: note all iPSCs are highly correlated. Hierarchical clustering using the complete linkage method and Euclidean distance from all loci (autosomes and sex chromosomes) for (e) methylation data (n = 455,910) and (f) gene expression data (n = 11,054).
(PDF)

S9 Fig Principal components analysis (PCA). Results of PCA on (a) methylation levels and (b) gene expression levels, using only autosomal loci in the iPSC samples.
(PDF)

S10 Fig Heatmap of DM loci. A heatmap of methylation levels at loci DM between L-iPSC and F-iPSC (n = 197), ordered by genomic location.
(PDF)

S11 Fig DE tests in gene subsets. To confirm that the test to detect DE genes was not underpowered, we also tested for DE in subsets of genes most likely to be DE between L-iPSC and F-iPSC–genes that were identified as DE in the other contrasts tested. We found no enrichment of significant P-Values based on DE tests with these subsets; see QQ plot of P-Values considering DE tests between L-iPSCs and F-iPSCs using four distinct gene sets: all genes, only genes DE between LCL and fibroblasts, only genes DE between LCL and L-iPSCs, and only genes DE between fibroblasts and F-iPSCs.
(PDF)

S12 Fig Differential methylation with single L-iPSC replicate. A Venn diagram depicting differentially methylated (DM) loci identified at an FDR of 5% overlapping between different contrasts with only a single L-iPSC replicate from each individual. A general decrease in the number of DM loci is observed across all contrasts as limma models all the data together. Yet, a far more marked decrease in the number of DM loci is observed in contrasts containing L-iPSCs.
(PDF)

S13 Fig Proportion of variation analysis using only genes and CpGs with previous evidence of genetic associations. Proportion of variation explained by individual and cell type of origin for (a) methylation levels of CpGs with an meQTL and (b) gene expression levels of genes with an eQTL.
(PDF)

S1 Table Sample covariate table. PluriTest Result, 0 = not pluripotent, 1 = pluripotent; Cell Type, 1 = iPSC, 2 = LCL, 3 = Fibroblast; Origin, 1 = L-iPSC, 2 = F-iPSC, 3 = LCL, 4 = Fibroblast; Sex, 1 = male, 2 = female. Reprogramming batch 1 = 1/29/2014, 2 = 2/3/2014, 3 = 1/24/2014, 4 = 2/10/2014, 5 = 2/24/2014, and 6 = 3/26/2014.
(XLSX)

S2 Table Differential methylation results. Differential methylation results from all contrasts (a) between L-iPSCs and F-iPSCs, (b) L-iPSCs and LCL, (c) F-iPSCs and fibroblasts, and (d) LCLs and fibroblasts. The contrasts are polarized as written here–i.e. a negative T statistic indicates a decrease in methylation between the first cell type and the second.
(GZ) Click here for additional data file.

S3 Table Differential gene expression results. Differential gene expression results from all contrasts (a) between L-iPSCs and F-iPSCs, (b) L-iPSCs and LCL, (c) F-iPSCs and fibroblasts, and (d) LCLs and fibroblasts. The contrasts are polarized as written here–i.e. a negative T statistic indicates a decrease in expression between the first cell type and the second.
(ZIP)

S4 Table Differential gene expression results with single L-iPSC replicate. These are the only genes identified as DE (FDR 5%) when we used only one L-iPSC replicate per individual. The ‘number of times DE’ is the sum of times the gene had been classified as DE across all 81 possible combinations of comparisons involving a single replicate.
(XLSX)

S5 Table PCR primers and conditions. PCR primers and conditions for all PCRs used; TM = melting temperature. Cycling conditions were per manufacturers recommendations and more specifically 30 cycles of the following: 94°C for 15 seconds, 58°C for 20 seconds, and 68°C for 30 seconds (OneTaq DNA Polymerase, NEB).
(XLSX)
