# Supplementary figures and images for: Genetic Variation, Not Cell Type of Origin, Underlies the Majority of Identifiable Regulatory Differences in iPSCs
Source: PLoS Genet. 2016 Jan 26;12(1):e1005793. doi: 10.1371/journal.pgen.1005793 (PMC4727884; doi:10.1371/journal.pgen.1005793)

# PluriTest

Pluripotency score

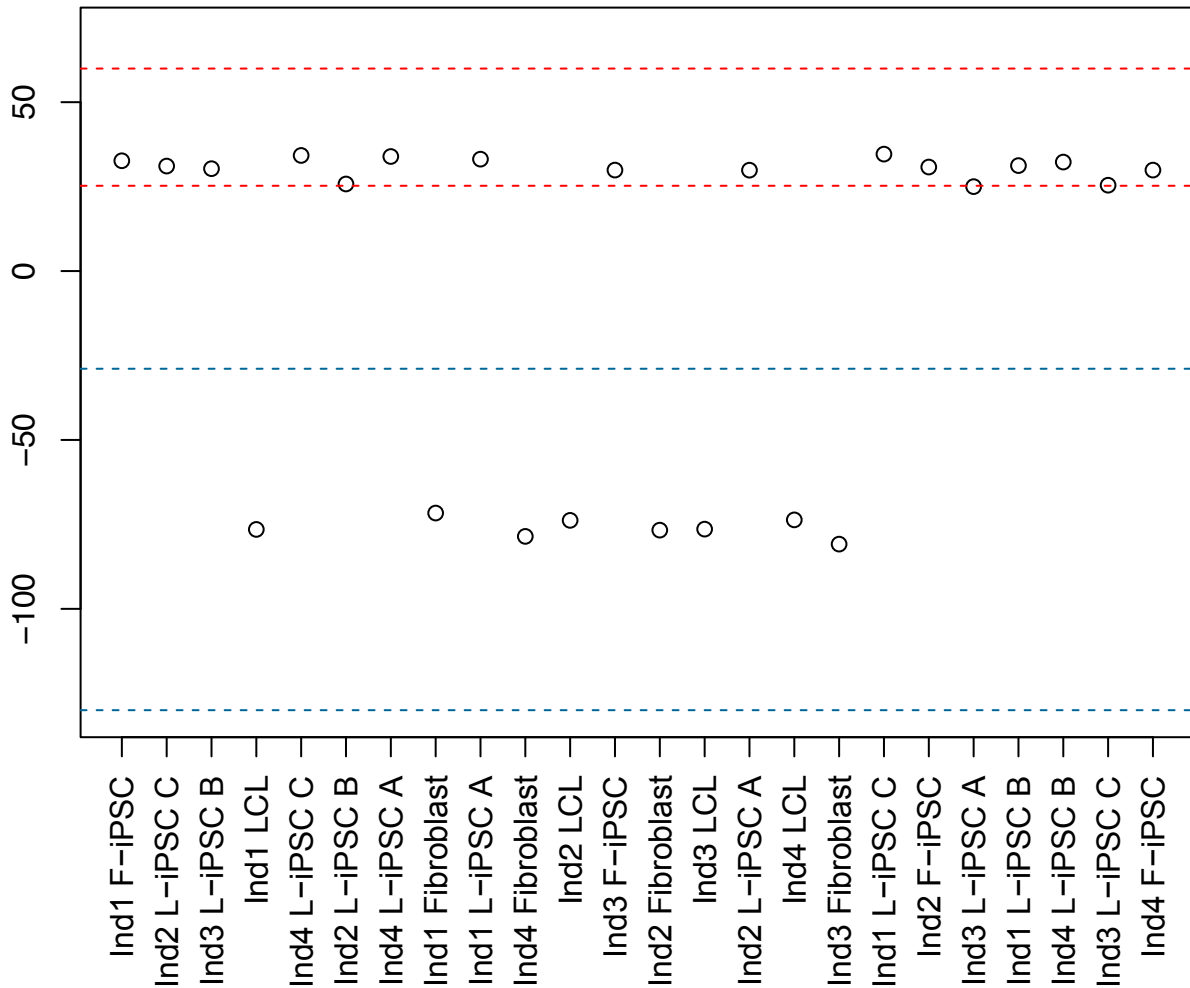

Supplement: S1 Fig — iPSC lines QC—PluriTest pluriscore results for all samples, showing all iPSC samples fall within the pluripotent threshold (red dashed lines). Additionally, all primary tissue samples fall within the non-iPSC cell type classification (blue dashed lines). (PDF) [file pgen.1005793.s001.pdf]

qPCR for canonical pluripotency transcription factors

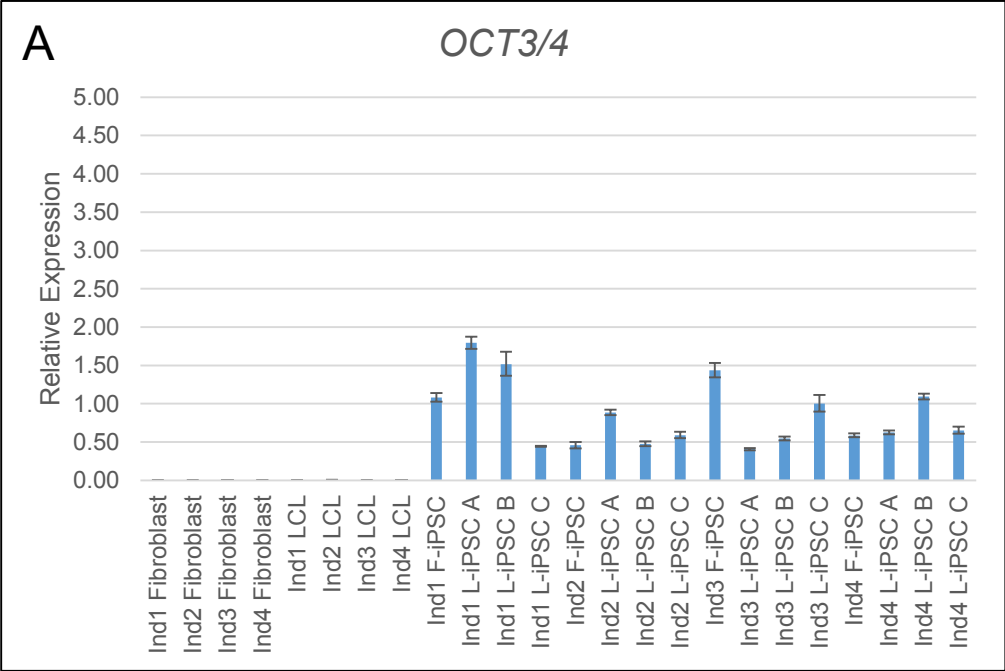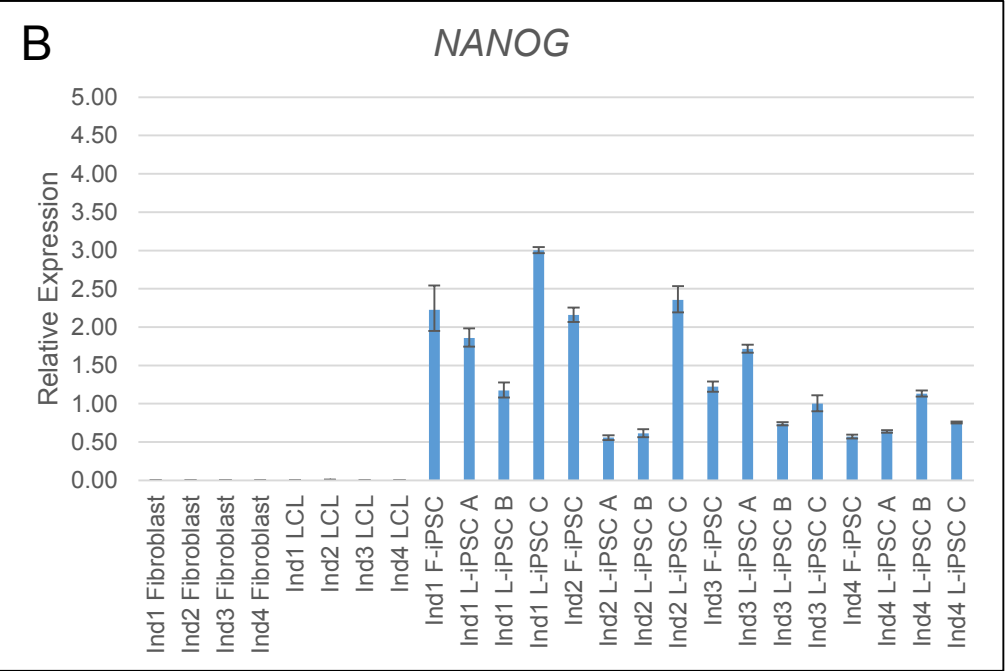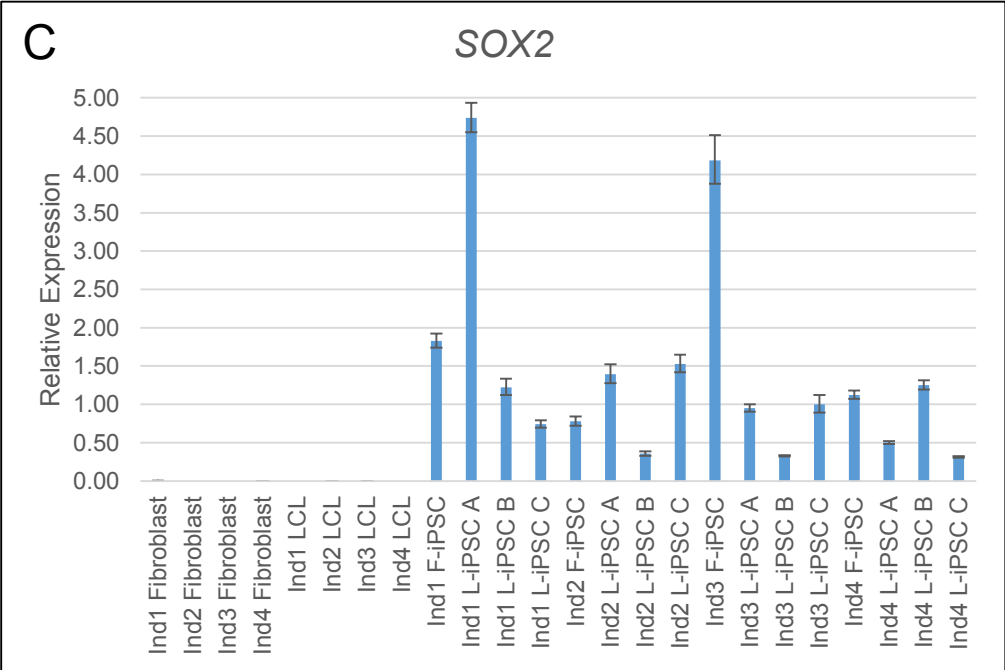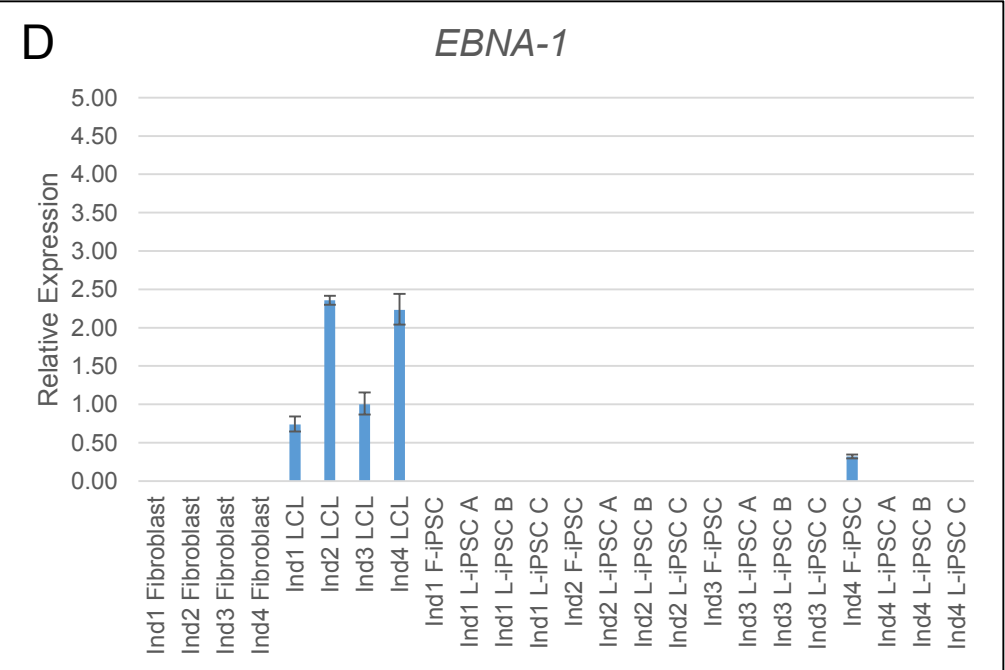

Supplement: S2 Fig — iPSC lines QC—Quantitative PCR (qPCR) of pluripotency genes (a) OCT3/4, (b) NANOG, and (c) SOX2 normalized on randomly selected Ind3 L-iPSC C. Relative expression is the RQ value with respect to GAPDH expression, with error bars representing the calculated min and max RQ value. All iPSC lines show endogenous expression of these pluripotency genes. (d) Expression of EBNA-1, a required viral gene of Epstein-Barr virus (EBV), normalized on randomly selected Ind3 LCL. EBNA-1 expression could stem from either the reprogramming vectors or, in LCLs and L-iPSCs, expression of integrated genomic EBV. Ind4 F-iPSC shows low expression of EBNA-1 due to low retention of reprogramming vectors as confirmed in Supplementary Fig 3. This sample is kept for data analysis because all other QC measures are met and the sample is not an outlier in overall gene expression or DNA methylation. (PDF) [file pgen.1005793.s002.pdf]

A

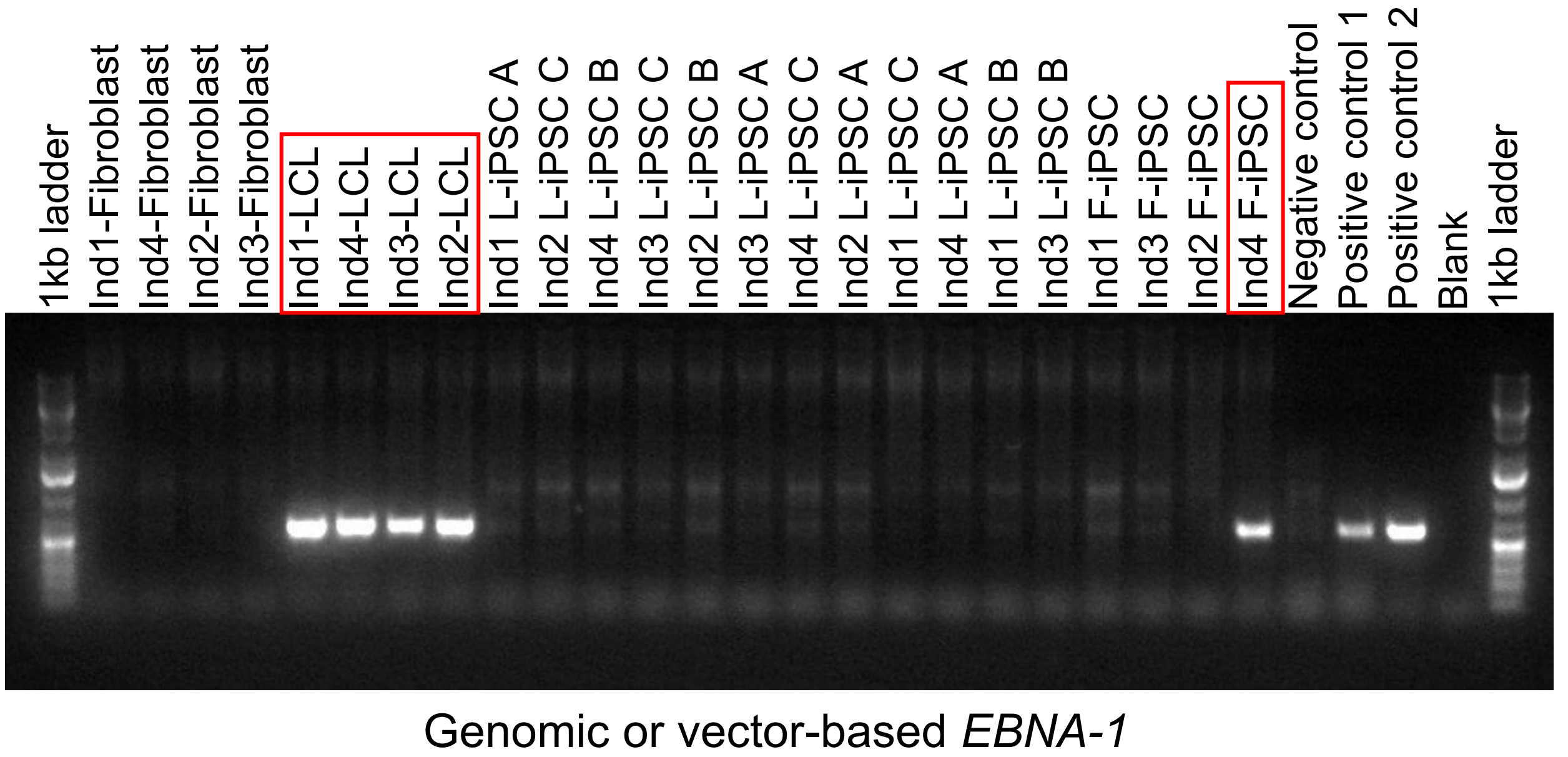

B

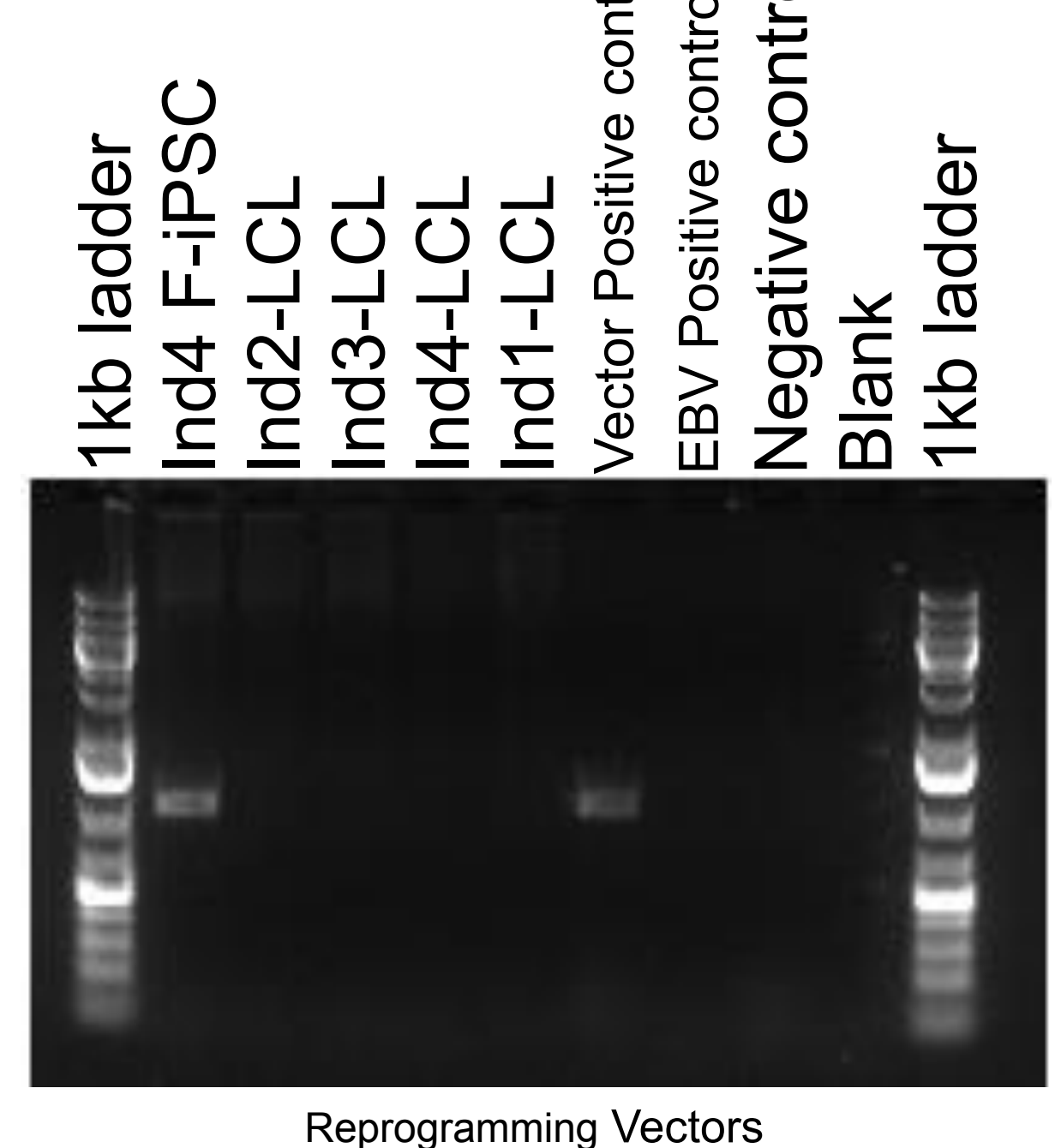

C

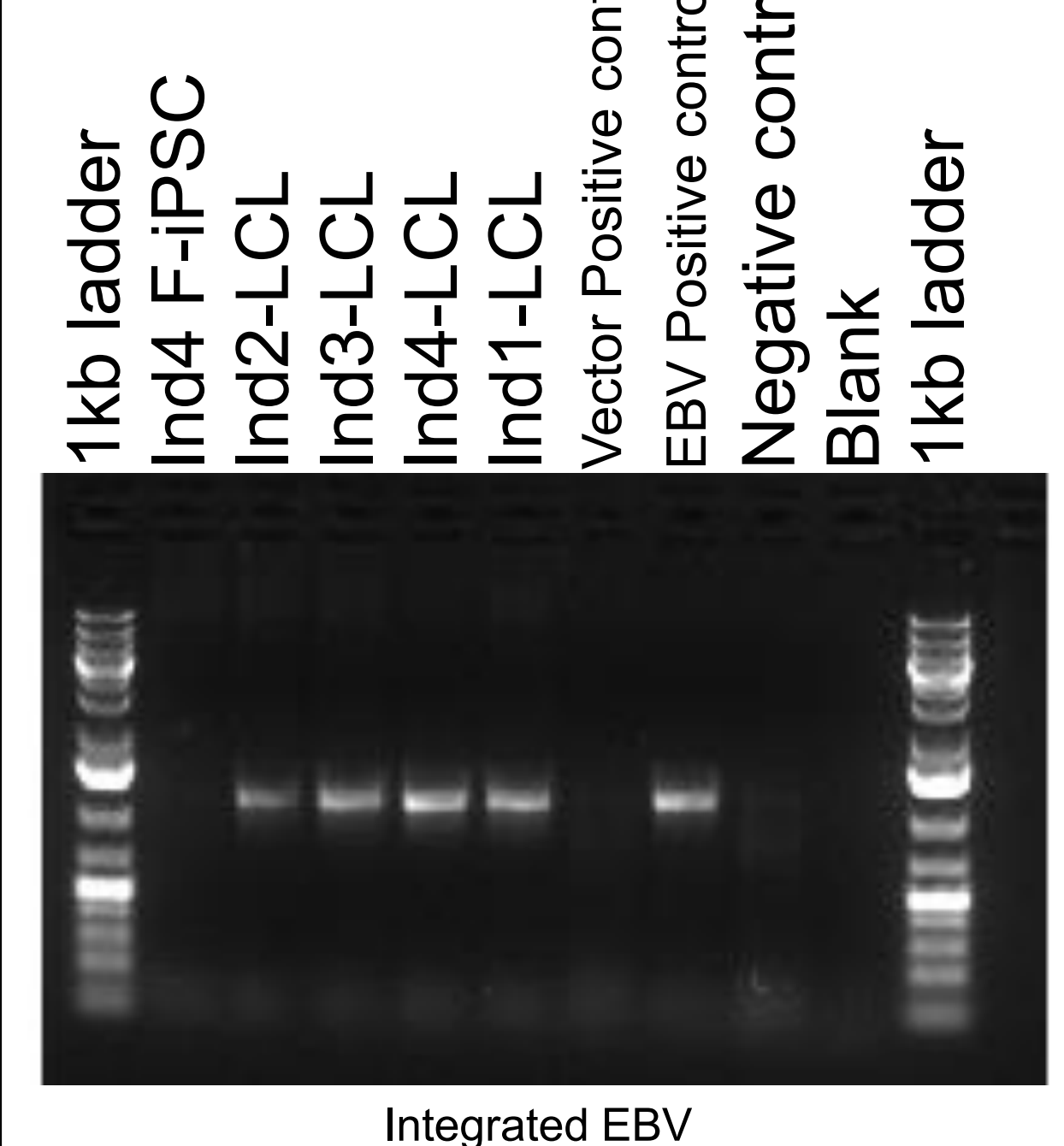

B

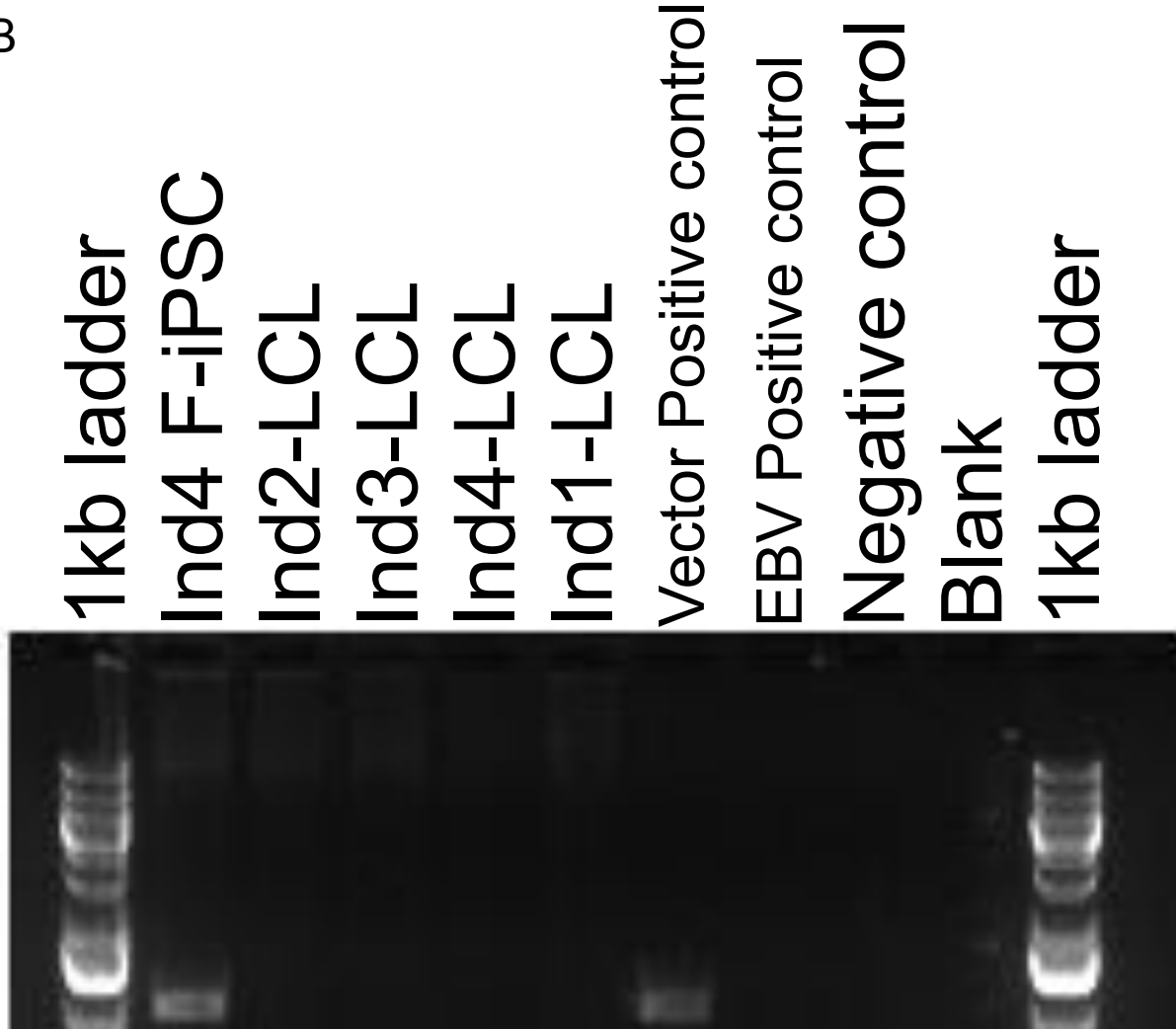

C

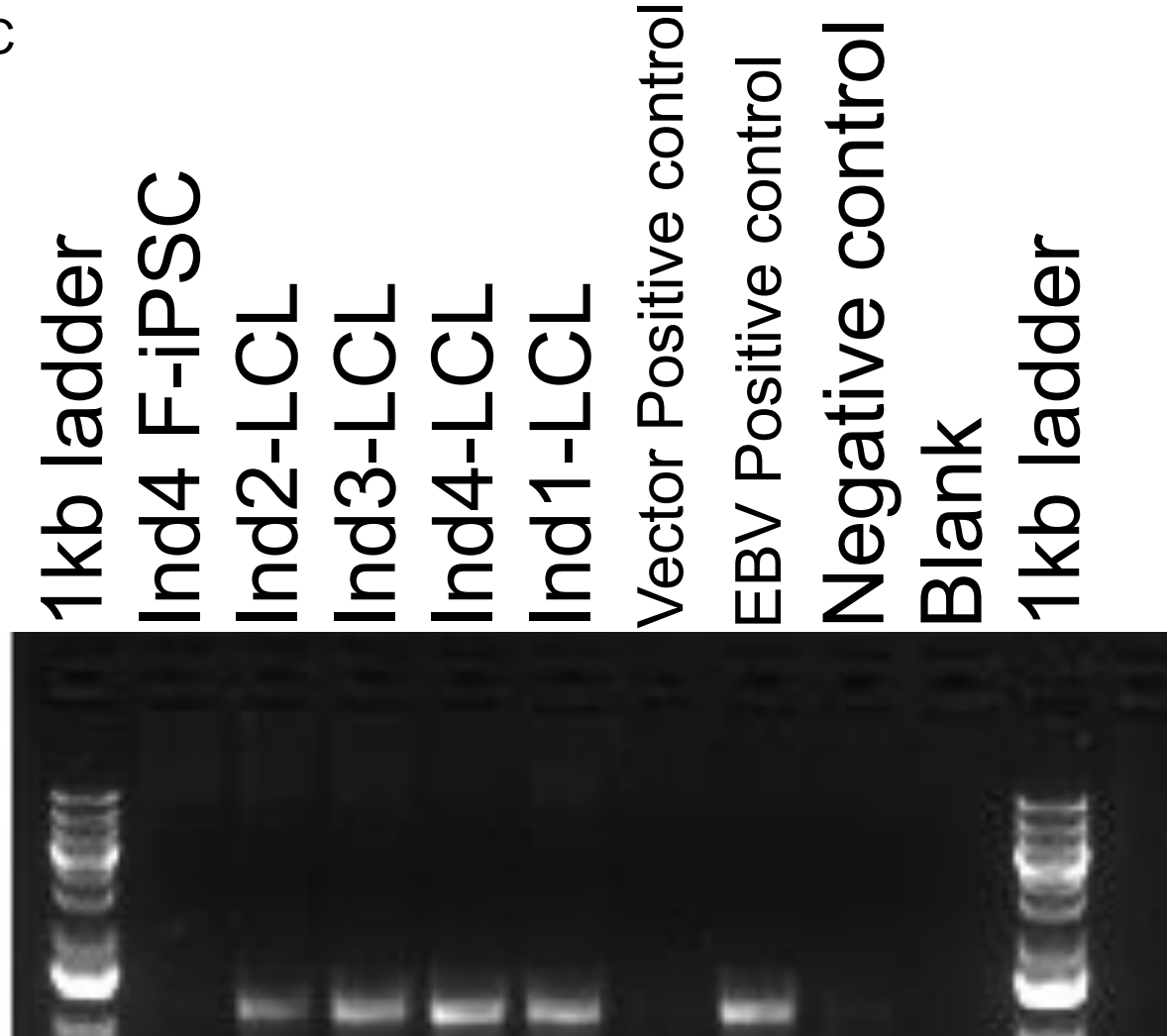

Genomic or vector-based *EBNA-1*

Supplement: S3 Fig — (a) PCR on DNA for presence or absence of EBV, both integrated and non-integrated (reprogramming vector based). All four LCLs showed the presence of EBV along with one iPSC line, Ind4 F-iPSC. Additional banding in the images is due to RNA in the sample. These five samples, highlighted by a red box, were taken forward for two additional PCRs. First, the five samples were tested for the presence of the reprogramming vectors (b), of which only Ind4 F-iPSC was positive. Lastly, the five samples were tested for EBV based on the presence of the LMP-2A sequence (c; an EBV gene not found on the reprogramming vector). All LCLs were positive for EBV, and the iPSC sample was not. (PDF) [file pgen.1005793.s003.pdf]

A

PCA of DNA methylation levles: iPSCs only

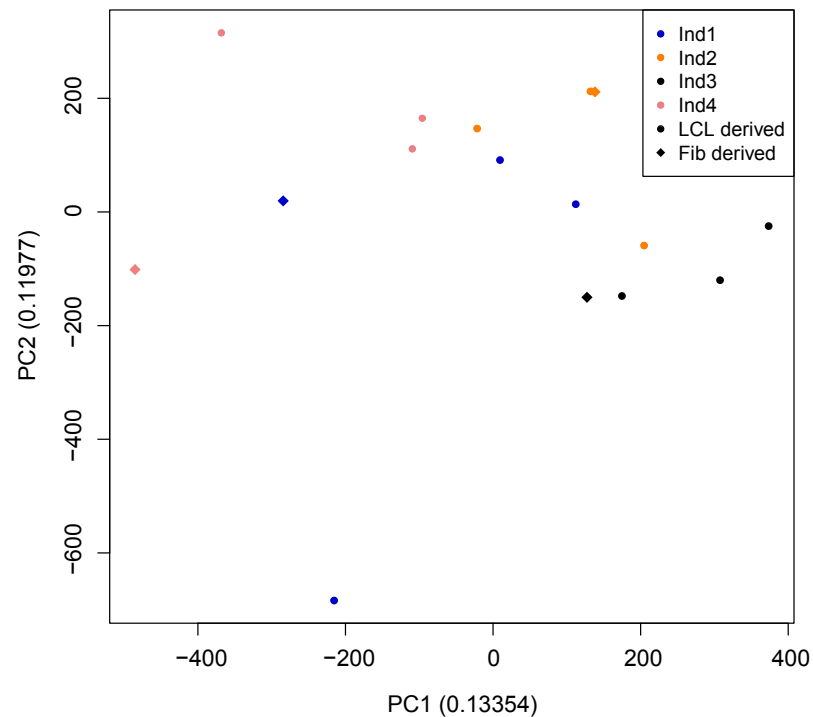

B

PCA of gene expression levels: iPSCs only

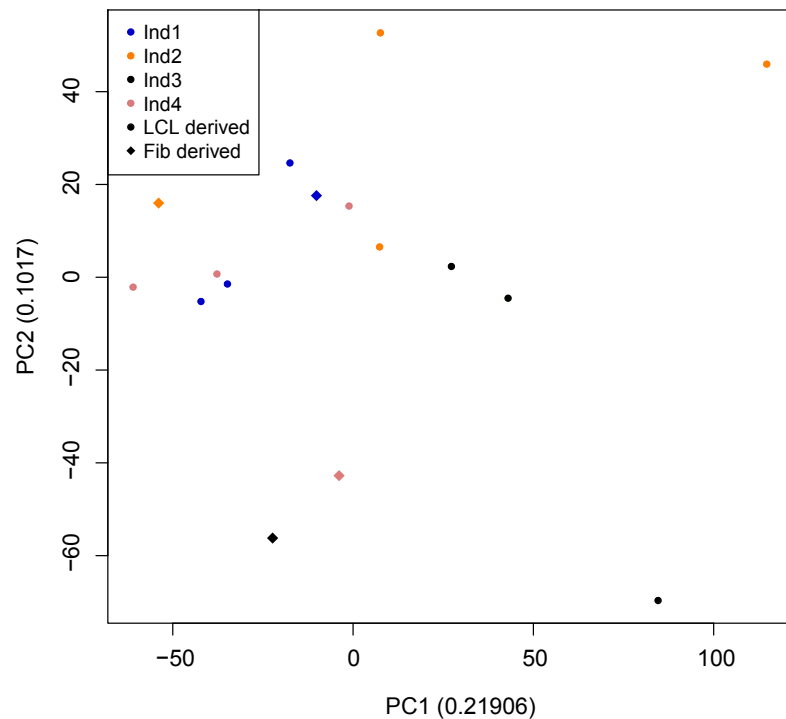

Supplement: S9 Fig — Results of PCA on (a) methylation levels and (b) gene expression levels, using only autosomal loci in the iPSC samples. (PDF) [file pgen.1005793.s009.pdf]

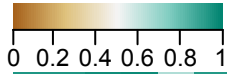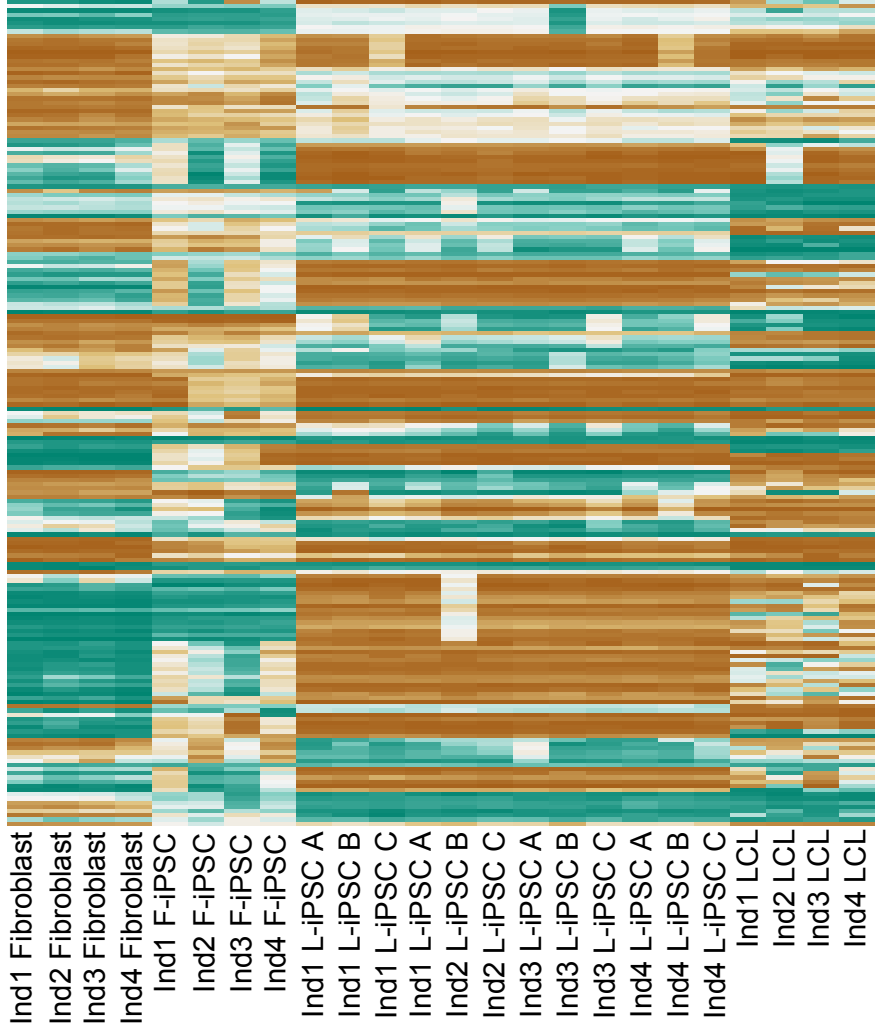

Supplement: S10 Fig — A heatmap of methylation levels at loci DM between L-iPSC and F-iPSC (n = 197), ordered by genomic location. (PDF) [file pgen.1005793.s010.pdf]

# QQ Plot by DE Gene Subsets

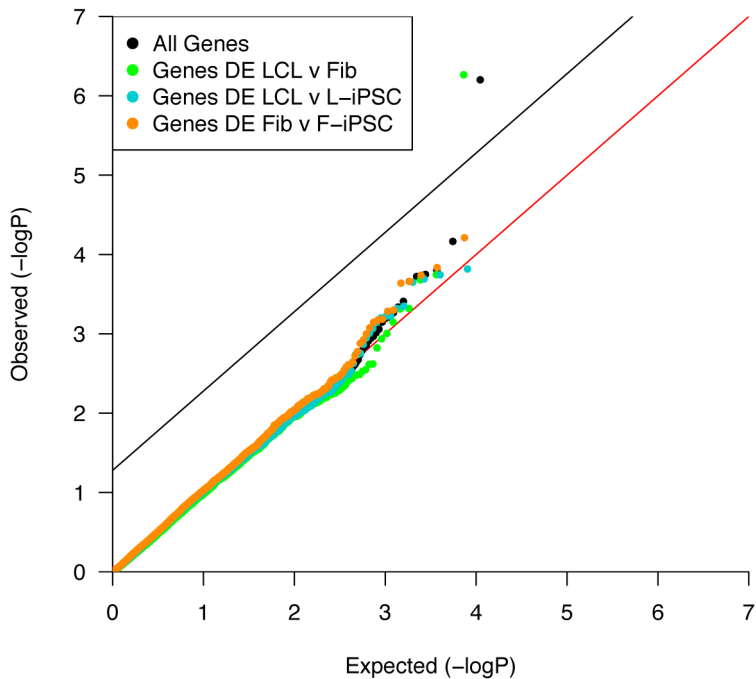

Supplement: S11 Fig — To confirm that the test to detect DE genes was not underpowered, we also tested for DE in subsets of genes most likely to be DE between L-iPSC and F-iPSC–genes that were identified as DE in the other contrasts tested. We found no enrichment of significant P-Values based on DE tests with these subsets; see QQ plot of P-Values considering DE tests between L-iPSCs and F-iPSCs using four distinct gene sets: all genes, only genes DE between LCL and fibroblasts, only genes DE between LCL and L-iPSCs, and only genes DE between fibroblasts and F-iPSCs. (PDF) [file pgen.1005793.s011.pdf]
