# Supplementary material for: Genetic Variation, Not Cell Type of Origin, Underlies the Majority of Identifiable Regulatory Differences in iPSCs
Source: PLoS Genet. 2016 Jan 26;12(1):e1005793. doi: 10.1371/journal.pgen.1005793 (PMC4727884; doi:10.1371/journal.pgen.1005793)
Supplement: S4 Fig — iPSC lines QC—Embryoid body (EB) formation from iPSC lines to validate the ability to differentiate into all three germ layers. The leftmost column (a) shows EBs stained with Nestin, a cytoplasmic stain for ectoderm in green and MAP2, a cytoplasmic stain for ectoderm in red. The center column (b) shows EBs stained with SMA, a cytoplasmic stain for mesoderm in green and again for MAP2 in red. The rightmost column (c) shows EBs stained with AFP, a cytoplasmic stain for endoderm in green and HNF3β, a nuclear stain for endoderm in red. All iPSC lines generated showed the ability to differentiate into all three germ layers. All imaging was done at 10x magnification and nuclei were stained blue with Hoechst. (PDF) [file pgen.1005793.s004.pdf]

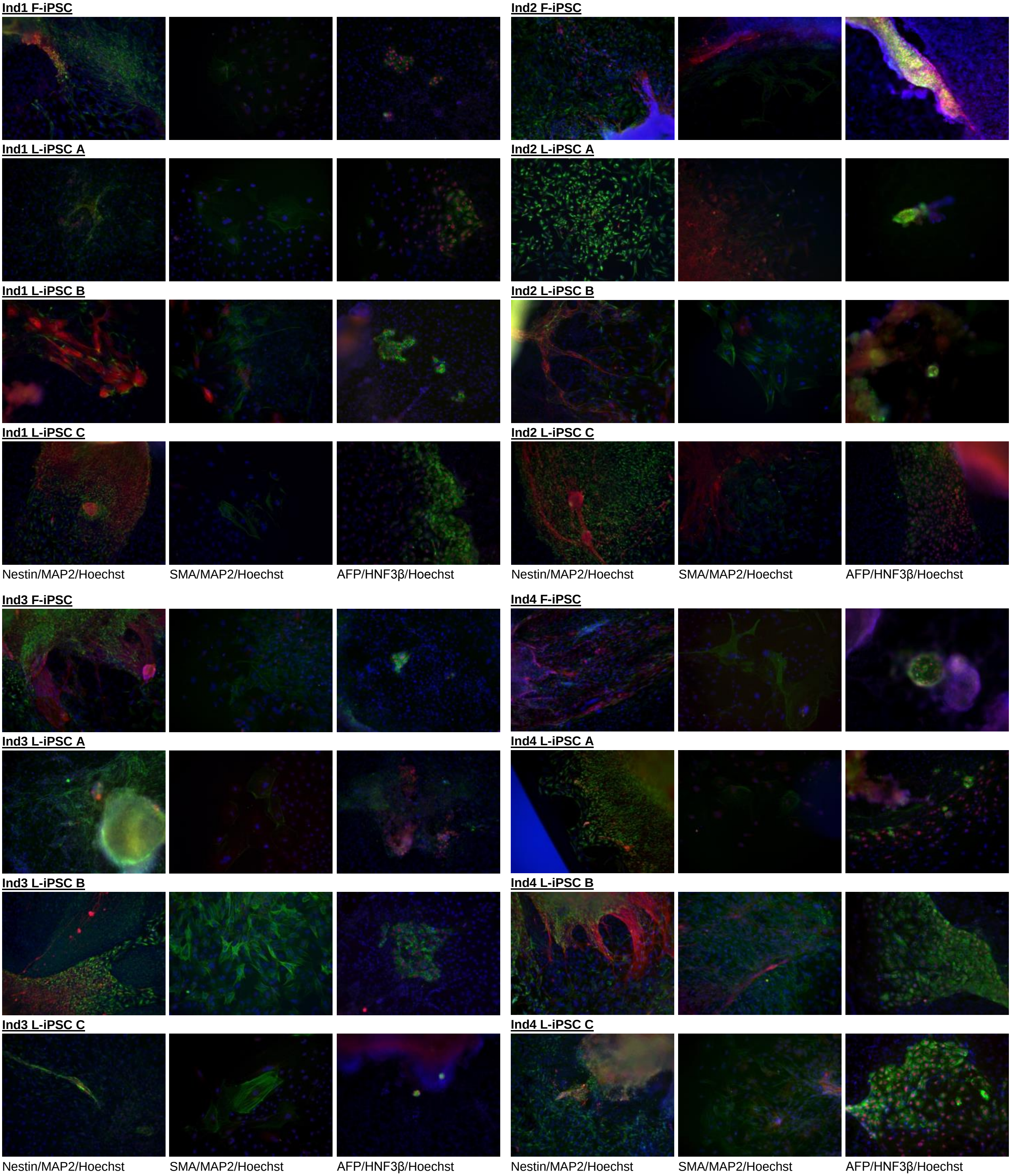
Ind1 F-iPSC
Ind2 F-iPSC
Ind1 L-iPSC A
Ind2 L-iPSC A
Ind1 L-iPSC B
Ind2 L-iPSC B
Ind1 L-iPSC C
Ind2 L-iPSC C
Nestin/MAP2/Hoechst
SMA/MAP2/Hoechst
AFP/HNF3β/Hoechst
Nestin/MAP2/Hoechst
SMA/MAP2/Hoechst
AFP/HNF3β/Hoechst
Ind3 F-iPSC
Ind4 F-iPSC
Ind3 L-iPSC A
Ind4 L-iPSC A
Ind3 L-iPSC B
Ind4 L-iPSC B
Ind3 L-iPSC C
Ind4 L-iPSC C
Nestin/MAP2/Hoechst
SMA/MAP2/Hoechst
AFP/HNF3β/Hoechst
Nestin/MAP2/Hoechst
SMA/MAP2/Hoechst
AFP/HNF3β/Hoechst
