# Supplementary material for: Genetic Variation, Not Cell Type of Origin, Underlies the Majority of Identifiable Regulatory Differences in iPSCs
Source: PLoS Genet. 2016 Jan 26;12(1):e1005793. doi: 10.1371/journal.pgen.1005793 (PMC4727884; doi:10.1371/journal.pgen.1005793)
Supplement: S5 Fig — Representative density plots of DNA methylation levels separated by type I and type II probes before and after SWAN Normalization. (PDF) [file pgen.1005793.s005.pdf]

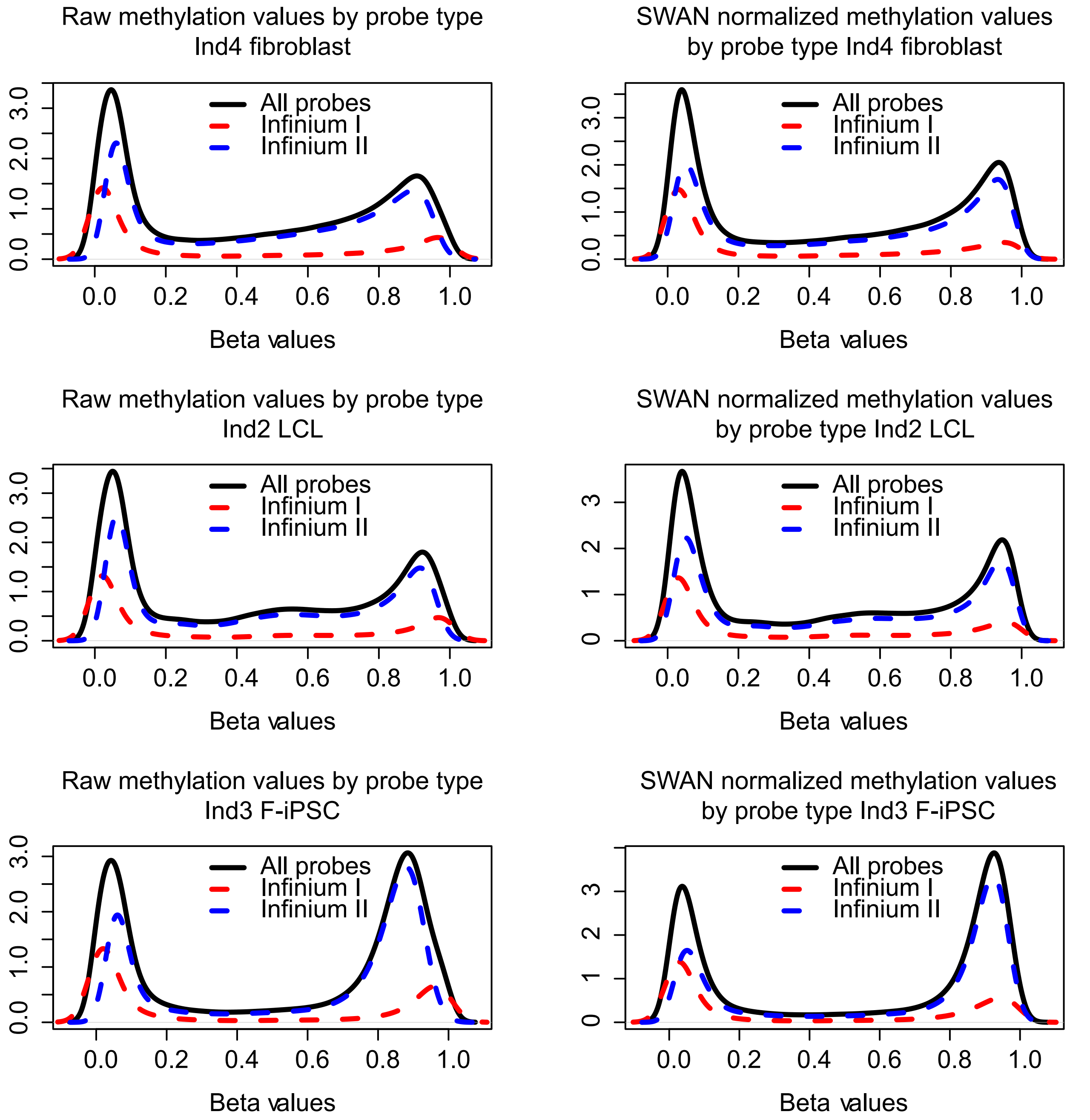
Raw methylation values by probe type
Ind4 fibroblast
SWAN normalized methylation values
by probe type Ind4 fibroblast
Raw methylation values by probe type
Ind2 LCL
SWAN normalized methylation values
by probe type Ind2 LCL
Raw methylation values by probe type
Ind3 F-iPSC
SWAN normalized methylation values
by probe type Ind3 F-iPSC
All probes
Infinium I
Infinium II
Beta values
0.0
0.2
0.4
0.6
0.8
1.0
0.0
1.0
2.0
3.0
0
1
2
3
