# Supplementary material for: Genetic Variation, Not Cell Type of Origin, Underlies the Majority of Identifiable Regulatory Differences in iPSCs
Source: PLoS Genet. 2016 Jan 26;12(1):e1005793. doi: 10.1371/journal.pgen.1005793 (PMC4727884; doi:10.1371/journal.pgen.1005793)
Supplement: S6 Fig — Methylation levels (Beta) (a) pre- and (b) post- quantile normalization. Quantile normalization was performed independently on the red and green color channels. Gene expression data (c) pre- and (d) post- quantile normalization. (PDF) [file pgen.1005793.s006.pdf]

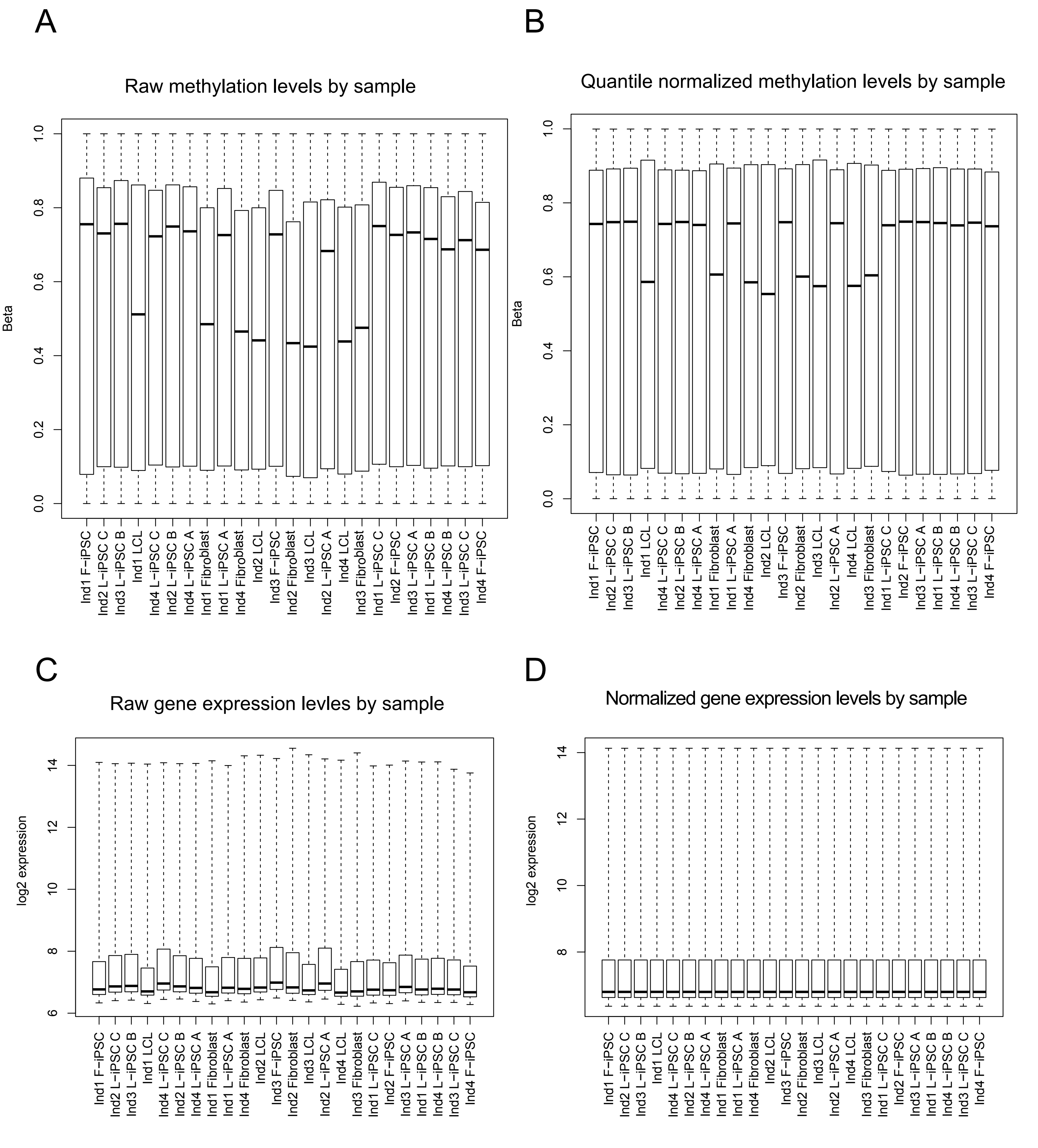
A
Raw methylation levels by sample
Beta
0.0
0.2
0.4
0.6
0.8
1.0
Ind1 F−iPSC
Ind2 L−iPSC C
Ind3 L−iPSC B
Ind1 LCL
Ind4 L−iPSC C
Ind2 L−iPSC B
Ind4 L−iPSC A
Ind1 Fibroblast
Ind1 L−iPSC A
Ind4 Fibroblast
Ind2 LCL
Ind3 F−iPSC
Ind2 Fibroblast
Ind3 LCL
Ind2 L−iPSC A
Ind4 LCL
Ind3 Fibroblast
Ind1 L−iPSC C
Ind2 F−iPSC
Ind3 L−iPSC A
Ind1 L−iPSC B
Ind4 L−iPSC B
Ind3 L−iPSC C
Ind4 F−iPSC
B
Quantile normalized methylation levels by sample
Beta
0.0
0.2
0.4
0.6
0.8
1.0
Ind1 F−iPSC
Ind2 L−iPSC C
Ind3 L−iPSC B
Ind1 LCL
Ind4 L−iPSC C
Ind2 L−iPSC B
Ind4 L−iPSC A
Ind1 Fibroblast
Ind1 L−iPSC A
Ind4 Fibroblast
Ind2 LCL
Ind3 F−iPSC
Ind2 Fibroblast
Ind3 LCL
Ind2 L−iPSC A
Ind4 LCL
Ind3 Fibroblast
Ind1 L−iPSC C
Ind2 F−iPSC
Ind3 L−iPSC A
Ind1 L−iPSC B
Ind4 L−iPSC B
Ind3 L−iPSC C
Ind4 F−iPSC
C
Raw gene expression levles by sample
log2 expression
6
8
10
12
14
Ind1 F−iPSC
Ind2 L−iPSC C
Ind3 L−iPSC B
Ind1 LCL
Ind4 L−iPSC C
Ind2 L−iPSC B
Ind4 L−iPSC A
Ind1 Fibroblast
Ind1 L−iPSC A
Ind4 Fibroblast
Ind2 LCL
Ind3 F−iPSC
Ind2 Fibroblast
Ind3 LCL
Ind2 L−iPSC A
Ind4 LCL
Ind3 Fibroblast
Ind1 L−iPSC C
Ind2 F−iPSC
Ind3 L−iPSC A
Ind1 L−iPSC B
Ind4 L−iPSC B
Ind3 L−iPSC C
Ind4 F−iPSC
D
Normalized gene expression levels by sample
log2 expression
8
10
12
14
Ind1 F−iPSC
Ind2 L−iPSC C
Ind3 L−iPSC B
Ind1 LCL
Ind4 L−iPSC C
Ind2 L−iPSC B
Ind4 L−iPSC A
Ind1 Fibroblast
Ind1 L−iPSC A
Ind4 Fibroblast
Ind2 LCL
Ind3 F−iPSC
Ind2 Fibroblast
Ind3 LCL
Ind2 L−iPSC A
Ind4 LCL
Ind3 Fibroblast
Ind1 L−iPSC C
Ind2 F−iPSC
Ind3 L−iPSC A
Ind1 L−iPSC B
Ind4 L−iPSC B
Ind3 L−iPSC C
Ind4 F−iPSC
