# Supplementary material for: Genetic Variation, Not Cell Type of Origin, Underlies the Majority of Identifiable Regulatory Differences in iPSCs
Source: PLoS Genet. 2016 Jan 26;12(1):e1005793. doi: 10.1371/journal.pgen.1005793 (PMC4727884; doi:10.1371/journal.pgen.1005793)

Probes with detectable gene expression by sample type

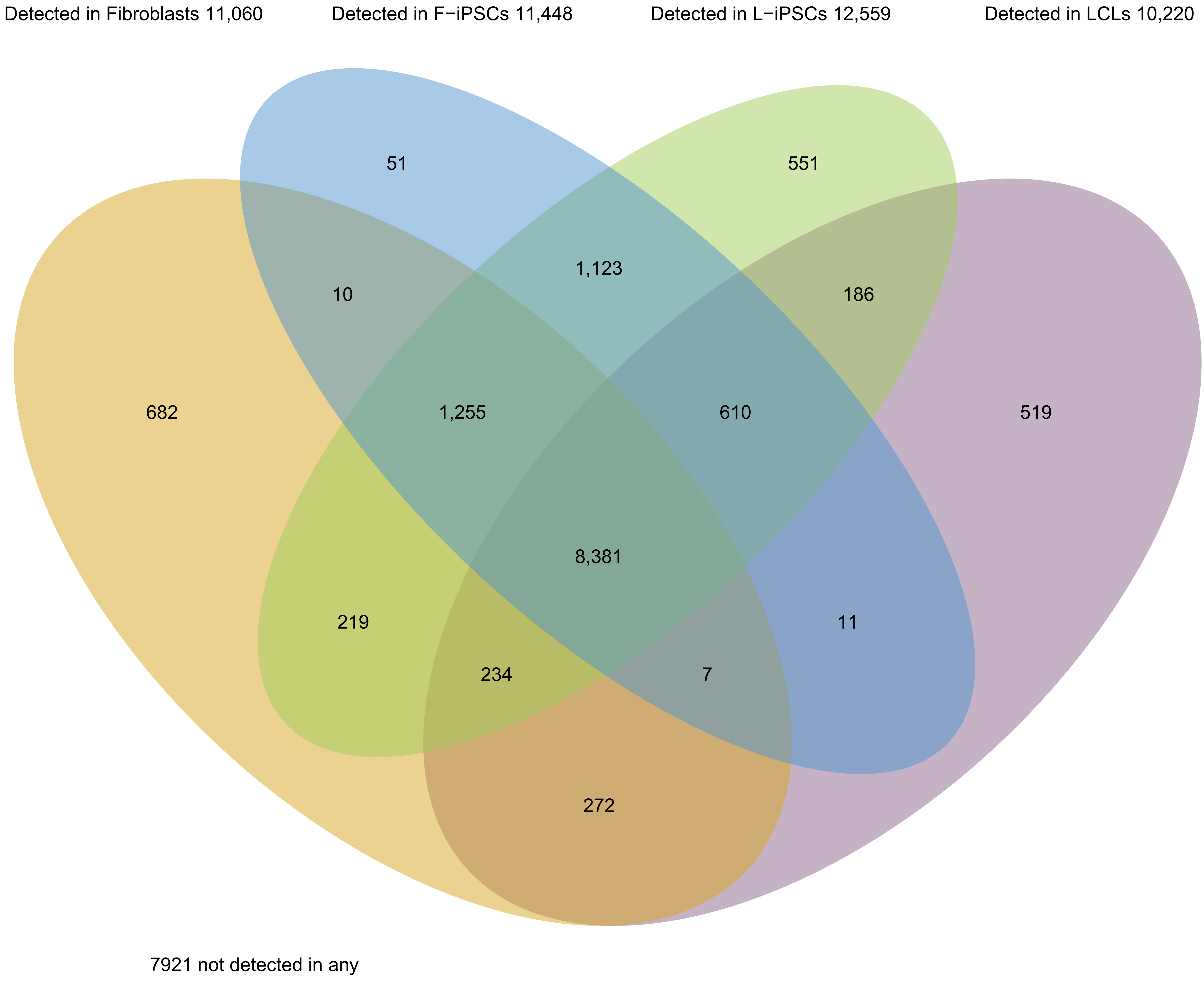

Supplement: S7 Fig — For 22,032 probes we defined a gene as expressed in a given sample if at least one probe mapping to it had a detection P-Value < 0.05. In the case of L-iPSCs, we defined a gene as expressed in an individual if any associated probes had a detection P-Value < 0.05 in at least one biological replicate. Using these criteria, we identified all genes expressed in at least three individuals in at least one cell type (n = 14,111 probes, associated with 11,054 genes). (PDF) [file pgen.1005793.s007.pdf]
