# Supplementary material for: Genetic Variation, Not Cell Type of Origin, Underlies the Majority of Identifiable Regulatory Differences in iPSCs
Source: PLoS Genet. 2016 Jan 26;12(1):e1005793. doi: 10.1371/journal.pgen.1005793 (PMC4727884; doi:10.1371/journal.pgen.1005793)
Supplement: S8 Fig — Hierarchical clustering using the complete linkage method and Euclidean distance from the 1,000 most variable autosomal iPSC loci for (a) methylation data and (b) expression data. Heatmap showing pairwise Pearson correlations between all samples for all loci (autosomes and sex chromosomes) (c) methylation data and (d) gene expression data: note all iPSCs are highly correlated. Hierarchical clustering using the complete linkage method and Euclidean distance from all loci (autosomes and sex chromosomes) for (e) methylation data (n = 455,910) and (f) gene expression data (n = 11,054). (PDF) [file pgen.1005793.s008.pdf]

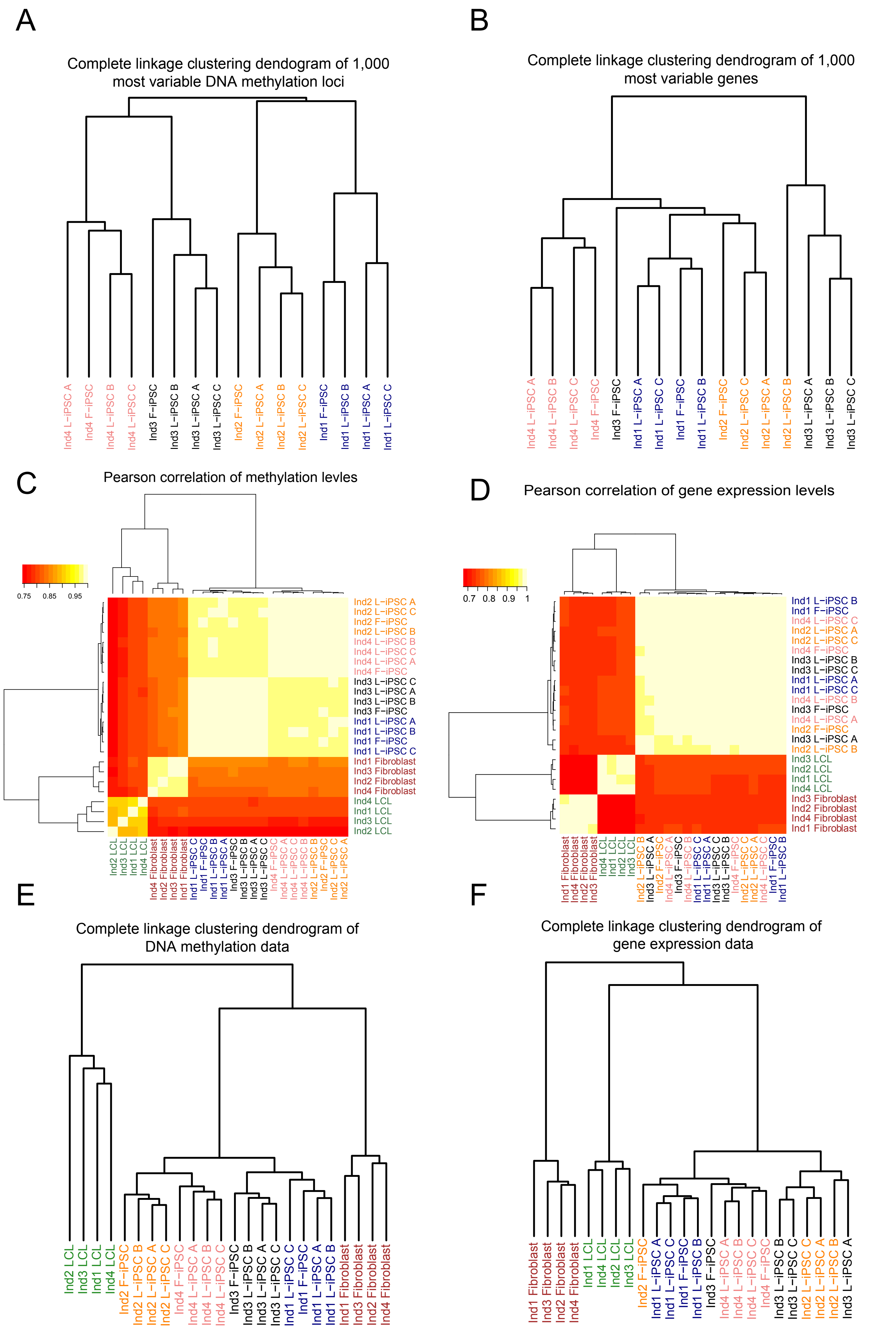
A
Complete linkage clustering dendogram of 1,000 most variable DNA methylation loci
Ind4 L−iPSC A
Ind4 F−iPSC
Ind4 L−iPSC B
Ind4 L−iPSC C
Ind3 F−iPSC
Ind3 L−iPSC B
Ind3 L−iPSC A
Ind3 L−iPSC C
Ind2 F−iPSC
Ind2 L−iPSC A
Ind2 L−iPSC B
Ind2 L−iPSC C
Ind1 F−iPSC
Ind1 L−iPSC B
Ind1 L−iPSC A
Ind1 L−iPSC C
B
Complete linkage clustering dendrogram of 1,000 most variable genes
Ind4 L−iPSC A
Ind4 L−iPSC B
Ind4 L−iPSC C
Ind4 F−iPSC
Ind3 F−iPSC
Ind1 L−iPSC A
Ind1 L−iPSC C
Ind1 F−iPSC
Ind1 L−iPSC B
Ind2 F−iPSC
Ind2 L−iPSC C
Ind2 L−iPSC A
Ind2 L−iPSC B
Ind3 L−iPSC A
Ind3 L−iPSC B
Ind3 L−iPSC C
C
Pearson correlation of methylation levles
0.75
0.85
0.95
Ind2 L−iPSC A
Ind2 L−iPSC C
Ind2 F−iPSC
Ind2 L−iPSC B
Ind4 L−iPSC B
Ind4 L−iPSC C
Ind4 L−iPSC A
Ind4 F−iPSC
Ind3 L−iPSC C
Ind3 L−iPSC A
Ind3 L−iPSC B
Ind3 F−iPSC
Ind1 L−iPSC A
Ind1 L−iPSC B
Ind1 F−iPSC
Ind1 L−iPSC C
Ind1 Fibroblast
Ind3 Fibroblast
Ind2 Fibroblast
Ind4 Fibroblast
Ind4 LCL
Ind1 LCL
Ind3 LCL
Ind2 LCL
Ind2 LCL
Ind3 LCL
Ind1 LCL
Ind4 LCL
Ind4 Fibroblast
Ind2 Fibroblast
Ind3 Fibroblast
Ind1 Fibroblast
Ind1 L−iPSC C
Ind1 F−iPSC
Ind1 L−iPSC B
Ind1 L−iPSC A
Ind3 F−iPSC
Ind3 L−iPSC B
Ind3 L−iPSC A
Ind3 L−iPSC C
Ind4 F−iPSC
Ind4 L−iPSC A
Ind4 L−iPSC C
Ind4 L−iPSC B
Ind2 L−iPSC B
Ind2 F−iPSC
Ind2 L−iPSC C
Ind2 L−iPSC A
D
Pearson correlation of gene expression levels
0.7
0.8
0.9
1
Ind1 L−iPSC B
Ind1 F−iPSC
Ind4 L−iPSC C
Ind2 L−iPSC A
Ind2 L−iPSC C
Ind4 F−iPSC
Ind3 L−iPSC B
Ind3 L−iPSC C
Ind1 L−iPSC A
Ind1 L−iPSC C
Ind4 L−iPSC B
Ind3 F−iPSC
Ind4 L−iPSC A
Ind2 F−iPSC
Ind3 L−iPSC A
Ind2 L−iPSC B
Ind3 LCL
Ind2 LCL
Ind1 LCL
Ind4 LCL
Ind3 Fibroblast
Ind2 Fibroblast
Ind4 Fibroblast
Ind1 Fibroblast
Ind1 Fibroblast
Ind4 Fibroblast
Ind2 Fibroblast
Ind3 Fibroblast
Ind4 LCL
Ind1 LCL
Ind2 LCL
Ind3 LCL
Ind2 L−iPSC B
Ind3 L−iPSC A
Ind2 F−iPSC
Ind4 L−iPSC A
Ind3 F−iPSC
Ind4 L−iPSC B
Ind1 L−iPSC C
Ind1 L−iPSC A
Ind3 L−iPSC C
Ind3 L−iPSC B
Ind4 F−iPSC
Ind2 L−iPSC C
Ind2 L−iPSC A
Ind4 L−iPSC C
Ind1 F−iPSC
Ind1 L−iPSC B
E
Complete linkage clustering dendrogram of DNA methylation data
Ind2 LCL
Ind3 LCL
Ind1 LCL
Ind4 LCL
Ind2 F−iPSC
Ind2 L−iPSC B
Ind2 L−iPSC A
Ind2 L−iPSC C
Ind4 F−iPSC
Ind4 L−iPSC A
Ind4 L−iPSC B
Ind4 L−iPSC C
Ind3 F−iPSC
Ind3 L−iPSC B
Ind3 L−iPSC A
Ind3 L−iPSC C
Ind1 L−iPSC C
Ind1 F−iPSC
Ind1 L−iPSC A
Ind1 L−iPSC B
Ind1 Fibroblast
Ind3 Fibroblast
Ind2 Fibroblast
Ind4 Fibroblast
F
Complete linkage clustering dendrogram of gene expression data
Ind1 Fibroblast
Ind3 Fibroblast
Ind2 Fibroblast
Ind4 Fibroblast
Ind1 LCL
Ind4 LCL
Ind2 LCL
Ind3 LCL
Ind2 F−iPSC
Ind1 L−iPSC A
Ind1 L−iPSC C
Ind1 F−iPSC
Ind1 L−iPSC B
Ind3 F−iPSC
Ind4 L−iPSC A
Ind4 L−iPSC B
Ind4 L−iPSC C
Ind4 F−iPSC
Ind3 L−iPSC B
Ind3 L−iPSC C
Ind2 L−iPSC C
Ind2 L−iPSC A
Ind2 L−iPSC B
Ind3 L−iPSC A
