# Supplementary material for: Genetic Variation, Not Cell Type of Origin, Underlies the Majority of Identifiable Regulatory Differences in iPSCs
Source: PLoS Genet. 2016 Jan 26;12(1):e1005793. doi: 10.1371/journal.pgen.1005793 (PMC4727884; doi:10.1371/journal.pgen.1005793)
Supplement: S12 Fig — A Venn diagram depicting differentially methylated (DM) loci identified at an FDR of 5% overlapping between different contrasts with only a single L-iPSC replicate from each individual. A general decrease in the number of DM loci is observed across all contrasts as limma models all the data together. Yet, a far more marked decrease in the number of DM loci is observed in contrasts containing L-iPSCs. (PDF) [file pgen.1005793.s012.pdf]

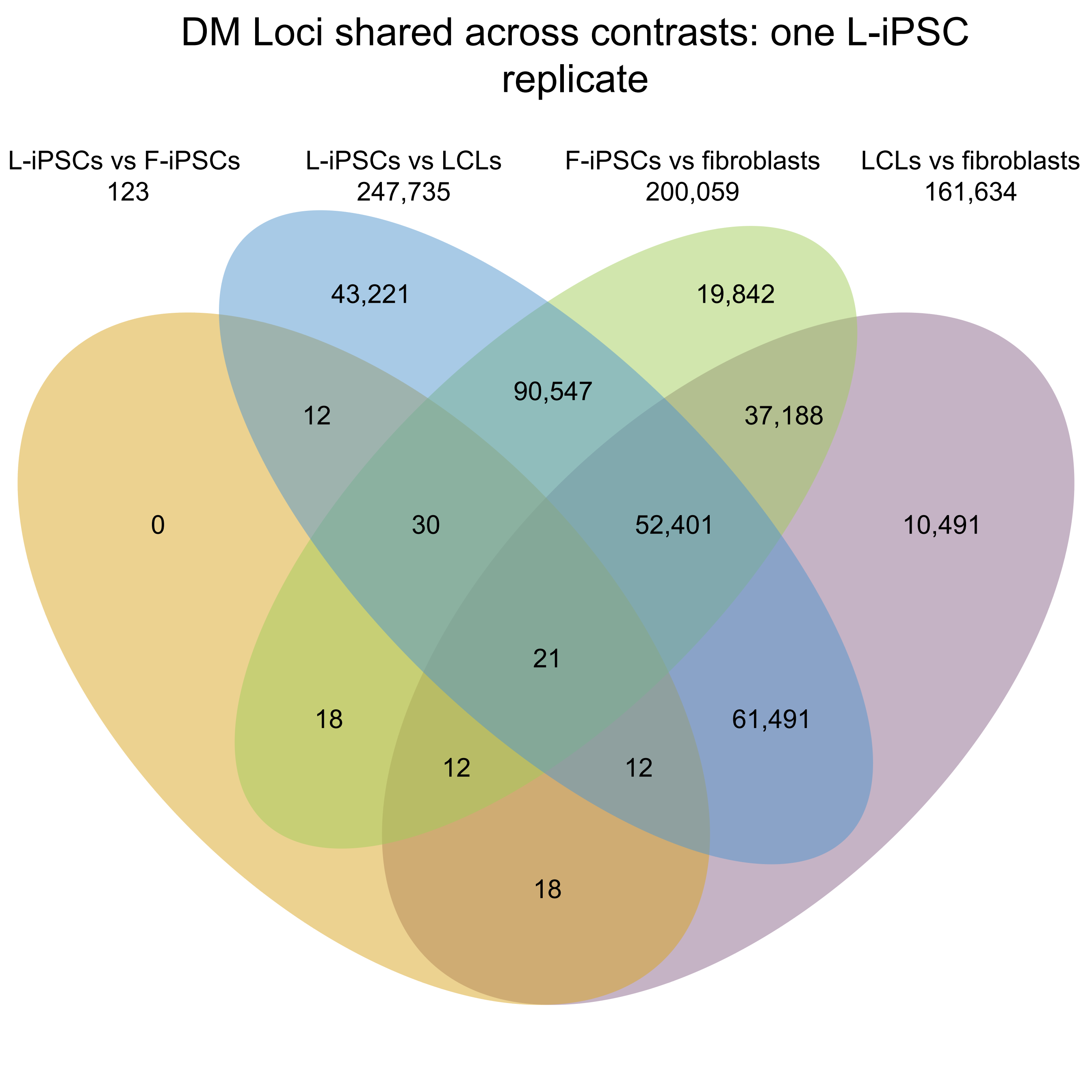
DM Loci shared across contrasts: one L-iPSC replicate
L-iPSCs vs F-iPSCs
123
L-iPSCs vs LCLs
247,735
F-iPSCs vs fibroblasts
200,059
LCLs vs fibroblasts
161,634
43,221
19,842
90,547
12
37,188
0
30
52,401
10,491
21
18
61,491
12
12
18
