# Supplementary material for: Genetic Variation, Not Cell Type of Origin, Underlies the Majority of Identifiable Regulatory Differences in iPSCs
Source: PLoS Genet. 2016 Jan 26;12(1):e1005793. doi: 10.1371/journal.pgen.1005793 (PMC4727884; doi:10.1371/journal.pgen.1005793)
Supplement: S13 Fig — Proportion of variation explained by individual and cell type of origin for (a) methylation levels of CpGs with an meQTL and (b) gene expression levels of genes with an eQTL. (PDF) [file pgen.1005793.s013.pdf]

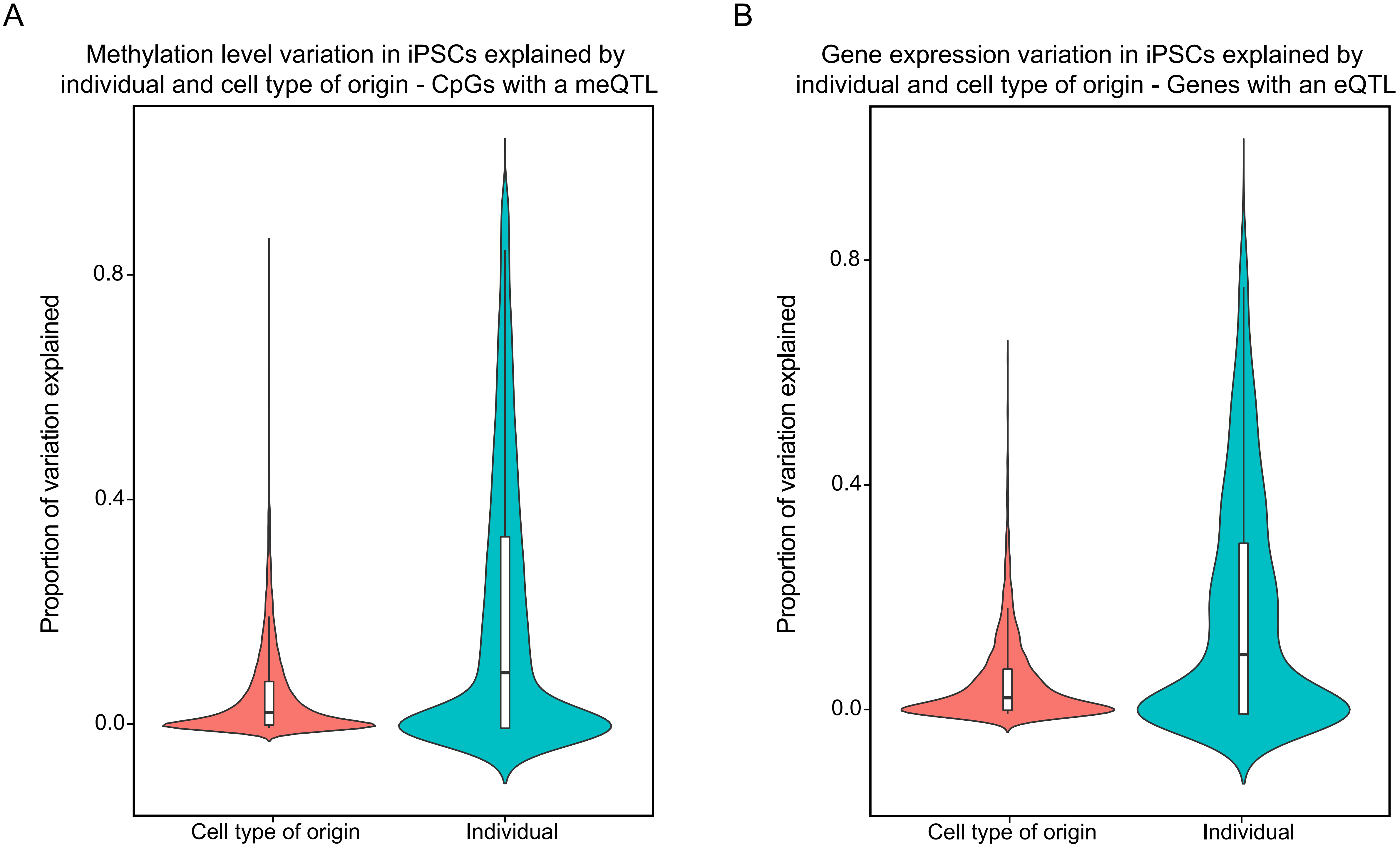
A
Methylation level variation in iPSCs explained by individual and cell type of origin - CpGs with a meQTL
Proportion of variation explained
0.8
0.4
0.0
Cell type of origin
Individual
B
Gene expression variation in iPSCs explained by individual and cell type of origin - Genes with an eQTL
Proportion of variation explained
0.8
0.4
0.0
Cell type of origin
Individual
